# De Novo Mutations in DENR Disrupt Neuronal Development and Link Congenital Neurological Disorders to Faulty mRNA Translation Re-initiation

**DOI:** 10.1016/j.celrep.2016.04.090

**Published:** 2016-05-26

**Authors:** Matilda A. Haas, Linh Ngo, Shan Shan Li, Sibylle Schleich, Zhengdong Qu, Hannah K. Vanyai, Hayley D. Cullen, Aida Cardona-Alberich, Ivan E. Gladwyn-Ng, Alistair T. Pagnamenta, Jenny C. Taylor, Helen Stewart, Usha Kini, Kent E. Duncan, Aurelio A. Teleman, David A. Keays, Julian I.-T. Heng

**Affiliations:** 1 EMBL Australia, The Australian Regenerative Medicine Institute, Monash University, Clayton, VIC 3800, Australia; 2 The Harry Perkins Institute of Medical Research, QEII Medical Centre and Centre for Medical Research, the University of Western Australia, Nedlands, WA 6009, Australia; 3 German Cancer Research Center (DKFZ), Im Neuenheimer Feld 580, 69120 Heidelberg, Germany; 4 Center for Molecular Neurobiology (ZMNH), University Medical Center Hamburg-Eppendorf, Falkenried 94, 20251 Hamburg, Germany; 5 National Institute for Health Research Biomedical Research Centre, Wellcome Trust Centre for Human Genetics, Roosevelt Drive, Oxford OX3 7BN, UK; 6 Department of Clinical Genetics, Churchill Hospital, Old Road, Headington, Oxford OX3 7LE, UK; 7 Institute of Molecular Pathology, Dr Bohr-Gasse, Vienna 1030, Austria

## Abstract

Disruptions to neuronal mRNA translation are hypothesized to underlie human neurodevelopmental syndromes. Notably, the mRNA translation re-initiation factor DENR is a regulator of eukaryotic translation and cell growth, but its mammalian functions are unknown. Here, we report that *Denr* influences the migration of murine cerebral cortical neurons in vivo with its binding partner *Mcts1*, whereas perturbations to *Denr* impair the long-term positioning, dendritic arborization, and dendritic spine characteristics of postnatal projection neurons. We characterized de novo missense mutations in *DENR* (p.C37Y and p.P121L) detected in two unrelated human subjects diagnosed with brain developmental disorder to find that each variant impairs the function of DENR in mRNA translation re-initiation and disrupts the migration and terminal branching of cortical neurons in different ways. Thus, our findings link human brain disorders to impaired mRNA translation re-initiation through perturbations in DENR (OMIM: 604550) function in neurons.

## Highlights

• DENR is an important player in mammalian neurodevelopment
• DENR controls radial migration through its binding partner MCTS1
• Mutations in DENR influence neuronal positioning and neurodifferentiation
• This study links impaired mRNA translation re-initiation to neurological disorders

## Graphical Abstract

**Figure:**
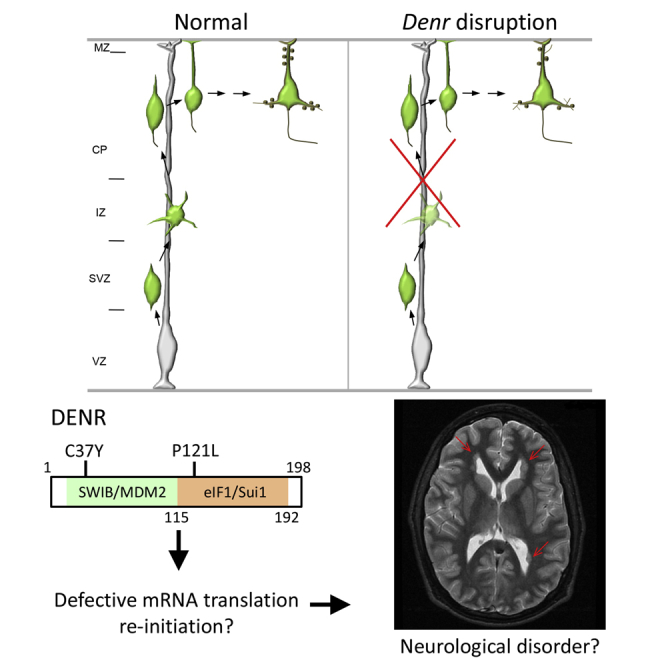

## Teaser

Haas et al. report that the expression of the mRNA translation re-initiation factor DENR is important for the radial positioning, dendritic branching, and dendritic spine properties of developing cerebral cortex neurons. Characterization of disease-associated mutations in *DENR* links impaired mRNA translation re-initiation to human neurological disorders.

## Introduction

Over the course of mammalian brain development, new neurons are generated from local progenitor zones and undergo migration before settling in their appropriate positions and forming functional circuits. Key to this process is the regulation of mRNA translation and intracellular protein synthesis in order for neurons to respond to environmental guidance cues for directional cell migration, axon outgrowth, dendritic arborization, and synaptic connectivity (Barnes and Polleux, 2009, Holt and Schuman, 2013). Defects in these early steps of neurodevelopment can result in the abnormal placement of neurons, impaired development of neural connections, and dysregulated synaptic signaling. Such impairments to nervous system development are increasingly recognized as important underlying causes of pediatric neurological conditions, such as epilepsy, intellectual disability, and autism spectrum disorder (Barkovich et al., 2005, Barkovich et al., 2012, Kelleher and Bear, 2008, Stoner et al., 2014, Wegiel et al., 2010). How exactly translational control contributes to specific aspects of neurodevelopment and disease remains poorly understood.

In eukaryotic cells, the translation of cellular mRNAs, normally bearing a 5′ cap structure (m^7^G) and a 3′poly(A) tail, occurs through a canonical process of initiation, elongation, termination, and ribosomal recycling (Hinnebusch and Lorsch, 2012). The importance for canonical mRNA translation in human neuronal development is reflected by the finding that genetic mutations in translation factors, such as eIF2γ and eIF2B, cause X-linked intellectual disability and leukoencephalopathy (a neurological disorder with vanishing white matter), respectively (Borck et al., 2012, Bugiani et al., 2010, Li et al., 2004). However, there are several non-canonical mechanisms for translation initiation of viral mRNAs in eukaryotic cells that do not bear a 5′ cap or possess neither a 5′ cap nor a 3′poly(A) tail (Firth and Brierley, 2012, Skabkin et al., 2010, Zinoviev et al., 2015). The translation of such mRNA types involves the functions of several eukaryotic proteins, such as the density regulated protein, known as DENR, and its obligate binding partner MCTS1 (Skabkin et al., 2010, Zinoviev et al., 2015). DENR is known to play important roles during mammalian translation, in the processes of ribosome recycling, in initiation on some viral mRNAs (Skabkin et al., 2010), and in termination-dependent re-initiation after long open reading frames (ORFs) on calicivirus mRNAs (Zinoviev et al., 2015). It was also recently reported that DENR regulates mRNA translation during *Drosophila* development through a mechanism involving translation re-initiation after a “strong upstream open reading frame” (stuORF) sequence (Schleich et al., 2014). However, despite recent findings for its requirement in *Drosophila* development (Schleich et al., 2014) as well as a recent study implicating its role in the control of circadian rhythms in mouse cells (Janich et al., 2015), the functions of DENR in mammalian neuronal development remain unknown.

In this study, we demonstrate a critical role for DENR in the development of cerebral cortical neurons. We performed a series of in utero electroporation experiments in mice and found that disruptions to *Denr* impair the migration of neurons within the embryonic cerebral cortex, and this is dependent on the interaction of DENR with its binding partner MCTS1. We find that perturbations to *DENR* disrupt the long-term positioning of cortical projection neurons within the postnatal mouse cerebral cortex, as well as their terminal differentiation and dendritic spine density and morphology. In addition, we have characterized de novo missense mutations to DENR in two unrelated human subjects diagnosed with autism spectrum disorder. We find that substitution mutations to DENR impair its radial migration functions in vivo. Furthermore, we demonstrate that the presence of DENR substitutions is detrimental to the development and synaptic connectivity of cerebral cortical neurons. Our cell-based reporter assays additionally demonstrate that these substitution mutations disrupt mRNA translation initiation in different ways. Taken together, our studies highlight a critical function for DENR in neuronal development and suggest that *DENR* substitution mutations may be a causative factor for brain developmental disorder in humans.

## Results

### Denr Promotes Cell Migration within the Developing Mouse Cerebral Cortex through Mcts1

Recent functional studies in *Drosophila* identified Denr as a regulator of mRNA translation and tissue morphogenesis (Schleich et al., 2014). In humans, a de novo missense (c.110G > A) mutation resulting in a p.C37Y substitution to DENR was identified in a patient diagnosed with autism spectrum disorder (Neale et al., 2012), a neurodevelopmental disorder that is associated with disruptions to cortical neuron positioning, dendritogenesis, and synaptogenesis (Barkovich et al., 2005, Barkovich et al., 2012, Kelleher and Bear, 2008, Stoner et al., 2014, Wegiel et al., 2010). To search for additional DENR mutations in patients with an autism-related diagnosis, we conducted whole-exome sequencing of a patient diagnosed with Asperger syndrome and associated epilepsy to identify a de novo missense (c.362C > T, NM_003677) mutation that results in a p.P121L substitution (Table S1). No other de novo mutations were identified in the exome. Structural MRI studies of this individual revealed bilateral frontal periventricular nodal heterotopia, a hallmark of neuronal migration disorder (Barkovich et al., 2005, Barkovich et al., 2012). A search of publically available genomic databases (including Exome Aggregation Consortium [ExAC] version 0.3) revealed that the p.C37Y and p.P121L substitution variants are not present in the general human population, and neither substitution mutation has previously been implicated in human disease (data not shown). Thus, disruptions to *DENR* could lead to disorders of neuronal development.

**Figure 1:**
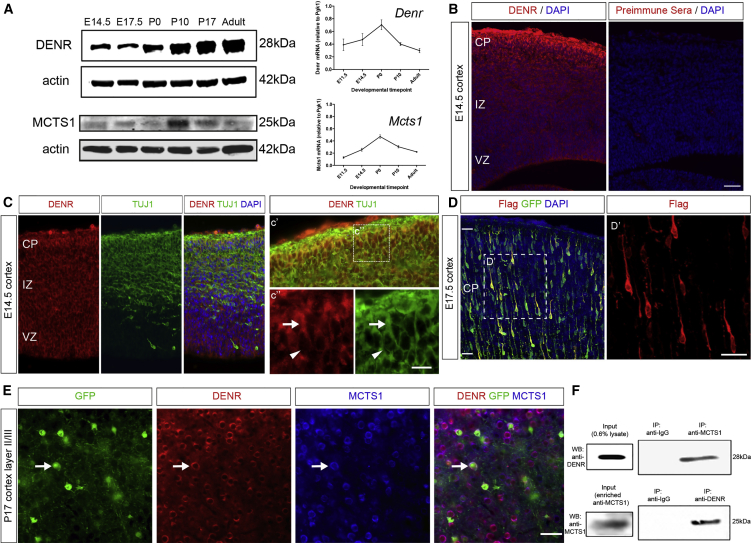
Denr and Mcts1 Expression during Mouse Brain Development (A) Immunoblotting reveals DENR and MCTS1 protein signal in mouse brain lysates collected from E14.5, E17.5, P0, P10, P17, and young adult (P30) brain tissue. qRT-PCR confirmed *Denr* and *Mcts1* mRNA expression surveyed from E11.5 through to P30 (a.u.; expression relative to *Pgk1*). (B) Immunofluorescence studies with a DENR antibody reveal a signal throughout cells of the ventricular zone (VZ) and cortical plate (CP). DENR (red) immunoreactivity was also coincident with neurons labeled with the neuronal marker TUJ1 (green). (C) High-magnification images (C’ and C’’) reveal co-localization of DENR and TUJ1 in the perinuclear cytoplasm (arrowheads) as well as the leading process of CP neurons (arrows). (D) Immunolabeling studies of E14.5-born, GFP-labeled CP neurons within the E17.5 cortex, which overexpress FLAG-DENR, reveal its predominantly cytoplasmic distribution, including their leading process. (E) Immunostaining reveals DENR (red) and MCTS1 (blue) co-localization in GFP-labeled cortical neurons within layer II–IV of the P17 mouse cortex following electroporation at E14.5. (F) DENR was co-immunoprecipitated with an MCTS1 antibody in studies with neonatal P0 mouse brain lysate. A reciprocal experiment with DENR antibody also leads to co-immunoprecipitation of MCTS1. Control immunoprecipitations were performed with preimmunized sera. Input lanes for DENR (detected from 20 μg of lysate) and MCTS1 expression in brain lysate were used in both immunoprecipitation experiments. MCTS1 signals were obtained by immunoprecipitation using goat anti-MCTS1 antibody from 2 mg total protein in brain lysate. Graphs plot mean ± SEM. The scale bars represent (B) 100 μm, (C’’) 12.5 μm, (D’) 7.5 μm, and (E) 40 μm.

Denr requires its binding partner Mcts1 to exert its effects on mRNA translation (Schleich et al., 2014, Skabkin et al., 2010), and so we investigated the expression of both Denr and Mcts1 during mammalian neuronal development at the protein and mRNA level. In humans, *DENR* and *MCTS1* mRNAs are detected in fetal, newborn, and adult brain (Figures S1A and S1B; Forrest et al., 2014). In mice, Denr and Mcts1 are detected throughout mouse brain development through to young adulthood (postnatal day 30 [P30]), with Denr protein levels increasing with brain age, whereas a peak in Mcts1 protein levels was observed at P10 (Figure 1A). Immunofluorescence staining of the embryonic mouse embryonic day 14.5 (E14.5) cerebral cortex revealed prominent Denr immunoreactivity in cells of the germinal ventricular zone (VZ), as well as in newborn neurons of the intermediate zone (IZ) and cortical plate (CP), which co-label with the early postmitotic marker βIII-tubulin (TUJ1; Figures 1B and 1C). We analyzed the immunofluorescence signal within immature neurons at higher magnification to find that DENR is localized within the cytoplasm, including the leading process of CP neurons that undergo radial migration (Nadarajah et al., 2002, Nadarajah et al., 2003, Noctor et al., 2004; Figures 1C’ and 1C”). Consistent with this pattern of cellular distribution, we cloned an expression construct encoding FLAG epitope-tagged DENR and transduced CP neurons by electroporation and observed prominent immunoreactivity within the cytoplasm and leading process (Figure 1D). To demonstrate DENR expression in cortical neurons within the postnatal cortex, we performed in utero electroporation to label E14.5-born neurons with GFP expression and then carried out immunostaining of electroporated brains harvested at P17 when neurons have completed their migration (Figure 1E). In addition to DENR immunoreactivity detected in GFP-labeled neurons, co-labeling studies with MCTS1 antibody revealed prominent localization of both fluorescence signals. We performed co-immunoprecipitation studies with postnatal mouse brain lysate to confirm that DENR interacts with MCTS1 in vivo (Figure 1F). Thus, DENR and MCTS1 interact in murine cortical neurons and could play a role in neuronal development within the cerebral cortex.

**Figure 2:**
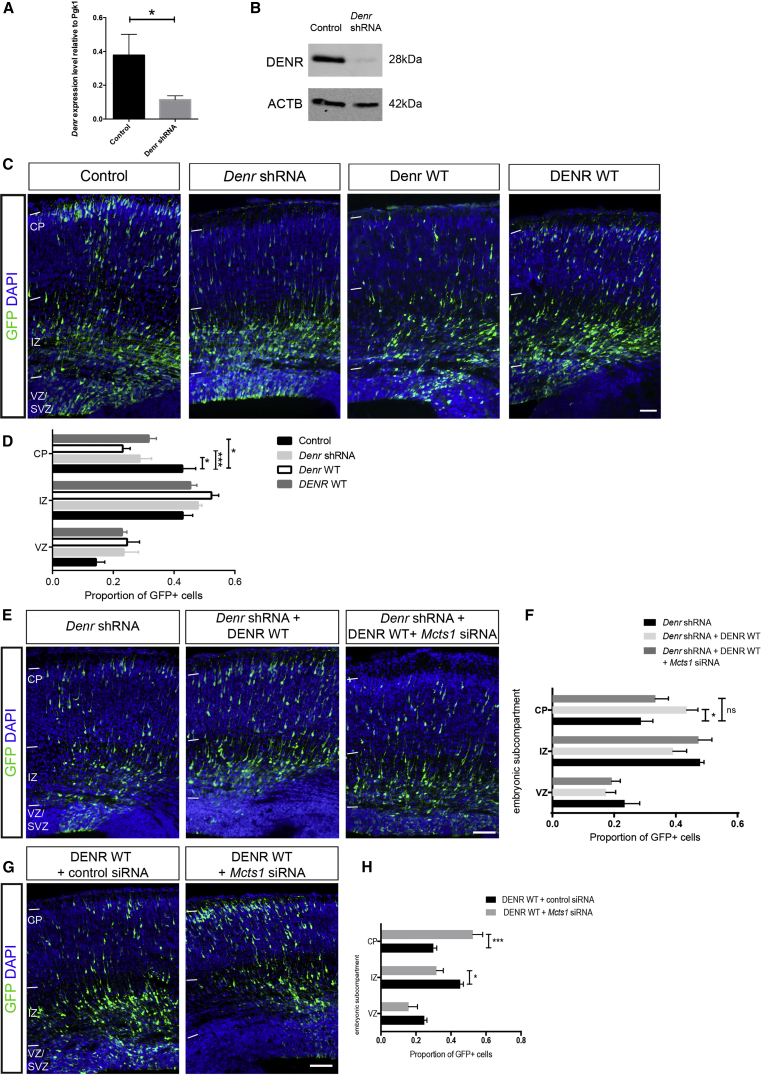
DENR Regulates Cell Migration in Concert with MCTS1 (A) qRT-PCR analysis of Neuro2A cells transiently transfected with either a control (non-targeting) shRNA construct or *Denr* shRNA construct revealed a significant decrease in *Denr* mRNA levels. (B) Parallel immunoblotting studies show depletion of Denr protein levels upon *Denr* shRNA treatment. (C) In utero electroporation studies of E14.5-labeled cortical cells within the E17.5 cortex following treatment with control (non-targeting shRNA vector with GFP-only vector), *Denr* shRNA (together with GFP-only vector), and forced expression of mouse Denr WT or human DENR WT (non-targeting shRNA vector with GFP expression vector comprising a *DENR* expression cassette). (D) *Denr* knockdown as well as overexpression of mouse or human DENR resulted in a significant reduction in the proportion of GFP+ cells in the CP of E17.5 cortices (control 0.426 ± 0.045; *Denr* shRNA 0.287 ± 0.039; Denr WT 0.231 ± 0.028; DENR WT 0.324 ± 0.027; two-way ANOVA F(4,33) = 3.980; p < 0.0096 with Bonferroni multiple comparison test; ^∗^p < 0.05; n = 3–5). (E and F) The defective migration of *Denr* shRNA-treated cells can be restored by co-delivery of human DENR WT, which is refractory to knockdown, but co-treatment with *Mcts1* siRNAs abolishes this effect (*Denr* shRNA 0.287 ± 0.039; *Denr* shRNA + DENR WT 0.435 ± 0.036; *Denr* shRNA + DENR WT + *Mcts1* siRNA 0.334 ± 0.042; two-way ANOVA F(2,30) = 2.897; p = 0.0386 with Bonferroni multiple comparison test; ^∗^p < 0.05; n = 3–5). (G and H) Whereas forced expression of DENR WT disrupts cell migration, co-delivery of *Mcts1* siRNAs abolishes this effect, observed as a significant increase in the proportion of GFP+ cells that arrive within CP (two-way ANOVA F(2,30) = 14.12; p < 0.0001; DENR WT + control siRNA 0.299 ± 0.02 versus DENR WT + *Mcts1* siRNAs 0.525 ± 0.056; Bonferroni multiple comparison test; ^∗∗∗^p < 0.005; n = 6), together with a concomitant reduction in the proportion of GFP+ cells within the IZ (*DENR WT* + control siRNA 0.453 ± 0.018 versus DENR WT + *Mcts1* siRNA 0.317 ± 0.04; Bonferroni multiple comparison test; ^∗^p < 0.05). Graphs plot mean ± SEM. The scale bars represent (C) 25 μm and (E and G) 50 μm.

Next, we performed in utero electroporation studies to determine whether DENR is important for the development of embryonic cortical neurons. We electroporated E14.5-born cortical cells with a bicistronic vector comprising a GFP expression cassette together with a *Denr* targeting short hairpin RNA (shRNA), which silences Denr expression in mouse Neuro2A cells (Figures 2A and 2B). We then analyzed the distribution of GFP-labeled cells within the brains of E14.5-electroporated embryos harvested 3 days later (at E17.5) to find that treatment with *Denr* shRNA resulted in a significant disruption of cortical cell migration compared to treatment with a control shRNA (Figures 2C and 2D). Treatment with *Denr* shRNA did not lead to a significant change in cortical progenitor proliferation as judged by immunolabeling for the mitosis marker pH3 (Figures S2A and S2B; p = 0.385 unpaired t test two-tailed; n = 6 control and 5 *Denr* shRNA-treated brains per condition) or their specification as apical progenitors or basal progenitors, as judged by co-labeling with Pax6 (Figure S2C) and Tbr2, respectively (Figure S2D). We performed experiments with a second *Denr* shRNA construct and observed comparable migration impairment (Figures S2E–S2G), suggesting that the radial migration functions for *Denr* are concentration sensitive. In support of this notion, we found that forced expression of mouse *Denr* (*Denr* wild-type [WT]) also impaired the migration of E14.5-derived cortical cells within the embryonic E17.5 cortex, indicative of a dominant, concentration-sensitive role. In parallel, we adopted a similar approach to find that overexpression of human *DENR* (*DENR WT*), in the presence of endogenous levels of mouse *Denr* expression, also impaired radial migration (Figures 2C and 2D). Neither knockdown of endogenous *Denr* nor forced expression of mouse *Denr* or human *DENR* led to precocious gliogenesis (immunostaining for glial fibrillary acidic protein [GFAP]) or excessive programmed cell death (visualized via immunostaining for the pro-apoptotic marker activated-caspase 3; Figures S2H and S2I), thus ruling out these factors as potential confounds. To further validate the specificity of *Denr* shRNA knockdown on cell migration, we performed a rescue experiment in which shRNA-treated cells were co-electroporated with mouse *Denr WT*, which is refractory to RNAi (Figure S2J). We found that the defective migration of *Denr* shRNA-treated cells was restored to levels resembling control treatment when co-treated with *Denr WT* (see Figures S2K and S2L; F(4,30) = 16; p < 0.001; two-way ANOVA). Similarly, the defective migration of *Denr* shRNA-treated cells could also be ameliorated by co-treatment of human *DENR WT*, which is refractory to RNAi (Figures 2E, 2F, S2G, and S2M). Of note, we found that the migration profile of *Denr* shRNA-treated cells rescued with mouse *Denr WT* was not significantly different to cells rescued with human *DENR WT* (Figure S2N; F(2,21) = 0.95; p = 0.403; two-way ANOVA). Thus, we find that appropriate levels of *Denr* are important for radial migration within the embryonic cortex, and mouse and human *DENR* are functionally equivalent in our in vivo assay for cortical neuron development.

We wanted to determine whether Denr required Mcts1 to promote cell migration in vivo. Using *Mcts1* small interfering RNAs (siRNAs) (Figure S2O), we found that knockdown of *Mcts1* disrupted the capacity for *DENR WT* to restore the defective migration of *Denr* shRNA-treated cells, because the profile of *Denr* shRNA + DENR WT + *Mcts1* siRNA-treated cells was not significantly different to *Denr* shRNA treatment (Figures 2E and 2F). In a reciprocal experiment, we found that the defective migration of *DENR*-overexpressing cells was abrogated upon co-delivery of *Mcts1* siRNA (Figures 2G and 2H), again suggesting that MCTS1 is an obligate partner to DENR in radial migration. However, knockdown of *Mcts1* alone does not disrupt migration, suggesting that remnant MCTS1 is likely sufficient for migration (Figures S2P and S2Q), whereas *Denr* disruption is sensitive to concurrent *Mcts1* knockdown by siRNAs in our migration assay. Also, the defective migration of *Denr* shRNA-treated cells is not exacerbated by co-treatment with *Mcts1* siRNAs (Figures S2R and S2S). Therefore, our data demonstrate that *Denr* and *Mcts1* coordinate radial migration within the embryonic cortex.

### Perturbations to Denr Expression Impair the Long-Term Positioning and Terminal Differentiation of Cerebral Cortical Neurons

**Figure 3:**
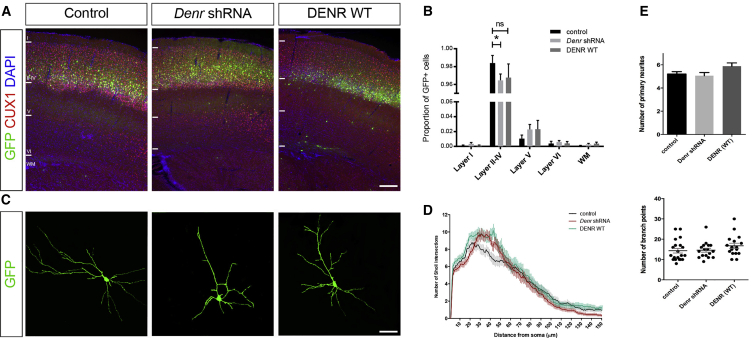
The Effects of Knockdown and Overexpression of DENR on the Long-Term Positioning and Dendritic Morphology of Postnatal Cortical Projection Neurons (A) Fluorescence immunostaining on coronal sections to study the positioning of GFP-labeled cells within the P17 postnatal cortex following in utero electroporation at E14.5. (B) Treatment with *Denr* shRNAs, but not DENR WT, led to a significant interaction in the positioning of neurons within the P17 cortex (two-way ANOVA F(8,80) = 1.095; p = 0.03754; n = 5–7), with only knockdown of *Denr* resulting in a significantly reduced proportion of GFP-positive cells in layers II–IV compared with control (control 0.984 ± 0.008; *Denr* shRNA 0.965 ± 0.007; DENR WT 0.968 ± 0.0153; ^∗^p < 0.05). (C) Representative confocal microscopy images of GFP-labeled, layer II/III cortical projection neurons from each condition. (D) Sholl analysis reveals differences in the branching complexity of neurons between conditions, with significant alterations in branching detected 30–50 μm from the cell body (see also Figures S3C–S3E). (E) Treatment with *Denr* shRNA or DENR WT did not significantly alter the number of primary neurites (two-way ANOVA F(2,51) = 2.846; p = 0.0673; n = 17–20 neurons per condition) or their branch points (two-way ANOVA F(2,51) = 1.358; p = 0.2662; n = 17–20 neurons per condition). Graphs plot mean ± SEM. The scale bars represent (A) 200 μm and (C) 25 μm.

Given that *Denr* immunoreactivity is prominent in projection neurons of the postnatal mouse cerebral cortex (Figure 1E), we extended our studies to investigate whether disruptions to *Denr* might affect the long-term positioning of E14.5-born neurons within the brains of treated mice harvested at P17, a time point by which their migration has concluded. We observed a significant interaction between the layer distribution of GFP+ neurons and *DENR* disruption (Figures 3A and 3B; two-way ANOVA F(8,80) = 1.095; p = 0.3754). Notably, knockdown of endogenous *Denr* led to a significant decrease in the proportions of GFP+ neurons in layers II–IV, together with a concomitant increase in neurons in layers V and VI. Forced expression of *DENR* WT did not significantly disrupt long-term positioning, despite the presence of scattered GFP+ cells in deeper layers (Figure S3A). We confirmed that these disruptions to *Denr* did not alter the expression of the neuronal fate specification marker CUX1 in GFP+ cells (Figure S3B). Thus, DENR is critical to the positioning of neurons within the postnatal cortex, but not their specification as cortical projection neurons.

To investigate whether DENR is important for the terminal differentiation of post-migratory neurons, we analyzed the dendritic morphology and dendritic spines properties of layer II/III cortical neurons following knockdown of endogenous *Denr* or overexpression of *DENR*. We combined GFP confocal microscopy with 3D image reconstruction to obtain images of GFP-labeled neurons and then performed Sholl analysis as a quantitative representation of the dendritic branching of neurons measured with increasing distance from the cell soma (Ngo et al., 2014). Our results show that both knockdown and overexpression led to increased dendritic complexity of neurons proximal (30–50 μm) to the cell body (Figures 3C, 3D, and S3C–S3E), but the numbers of primary neurites as well as their branch points were not significantly different across conditions (Figure 3E).

**Figure 4:**
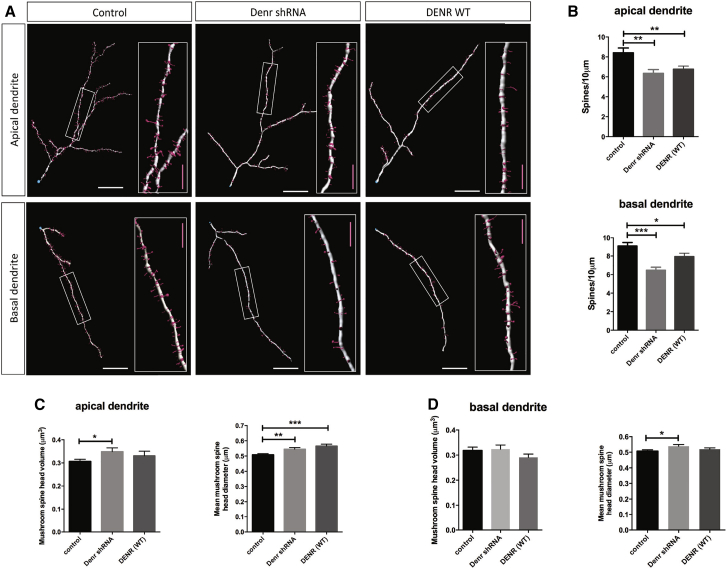
The Effects of DENR Perturbations on the Dendritic Spine Properties of Cortical Projection Neurons (A) Image reconstruction of confocal microscopy analyses to study dendritic spines from apical and basal dendrites of GFP-labeled neurons (see Experimental Procedures). (B) Treatment with *Denr* shRNA or forced expression of DENR WT led to a significant decrease in the density of dendritic spines along apical dendrites (control 8.417 ± 0.4771; *Denr* shRNA 6.350 ± 0.3839; DENR WT 6.768 ± 0.3091; one-way ANOVA F(2,44) = 7.598; p = 0.0015; n = 15–17; Bonferroni multiple comparisons ^∗∗^p < 0.01) as well as basal dendrites (control 9.122 ± 0.3651; *Denr* shRNA 6.476 ± 0.3171; DENR WT 7.938 ± 0.3751; one-way ANOVA F(2,52) = 13.82; p < 0.0001; Bonferroni multiple comparisons ^∗^p < 0.05; ^∗∗∗^p < 0.005). (C) Apical dendrite mushroom spine morphology. Treatment with *Denr* shRNA significantly increased the volume of mushroom-shaped spines compared to control (control 0.3070 ± 0.0089; *Denr* shRNA 0.3488 ± 0.01627; one-way ANOVA F(2,565) = 2.856; p = 0.0584; Bonferroni multiple comparisons ^∗^p < 0.05), whereas treatment with *Denr* shRNA and DENR WT significantly altered spine diameter (control 0.5082 ± 0.007397; *Denr* shRNA 0.5451 ± 0.01111; DENR WT 0.5655 ± 0.01259; one-way ANOVA F(2,565) = 9.076; p = 0.0001; Bonferroni multiple comparisons ^∗∗^p < 0.05; ^∗∗∗^p < 0.005). (D) Basal dendrite mushroom spine morphology. Whereas treatment conditions did not significantly affect the volume of mushroom-shaped spine heads on apical (two-way ANOVA F(2,565) = 2.856; p = 0.0584) and basal (two-way ANOVA F(2,468) = 1.209; p = 0.2994) dendrites, treatment with *Denr* shRNA significantly altered the spine diameter of apical dendrites (control 0.5066 ± 0.008637; *Denr* shRNA 0.5389 ± 0.01056; one-way ANOVA F(2,468) = 3.144; p = 0.0440; Bonferroni multiple comparisons ^∗^p < 0.05). Graphs plot mean ± SEM. The scale bars represent (A) 20 μm and (A insets) 5 μm.

Given the association between *DENR* mutations and autism spectrum disorder, a neurodevelopmental condition that is correlated with functional deficits in synaptic signaling (Chen et al., 2015), we analyzed dendritic spine density and spine morphologies of neurons in the context of *Denr* perturbations by combining high-power confocal microscopy with digital image reconstruction and morphometric analysis (Ngo et al., 2014; see Experimental Procedures). We performed separate analyses on apical and basal dendrites because it is known that both have different signaling properties and dendritic spine characteristics (Niblock et al., 2000). We found that knockdown as well as overexpression of *Denr* led to a significant reduction in dendritic spine densities on apical and basal dendrites (Figures 4A and 4B). In addition, we analyzed the shapes of dendritic spines and found that perturbations to *Denr* did not significantly alter the ratios of different spine types (classified as filopodia-, long-thin-, stubby-, and mushroom-shaped) on apical and basal dendrites (Figure S4). However, we were interested to investigate the properties of mushroom-shaped spines, known to represent mature synaptic contacts between neurons. We found that knockdown of endogenous *Denr* led to a significant increase in the diameter of mushroom-shaped spines on the apical as well as basal dendrites of cortical neurons, with *DENR* overexpression leading to a significant effect on spine head diameter on apical dendrites (Figures 4C and 4D). These results demonstrate that *Denr* perturbations impair the dendritic arborization, dendritic spine density, and spine morphology of cortical projection neurons.

### Substitution Mutations to DENR Impair Its Neuronal Differentiation Functions

**Figure 5:**
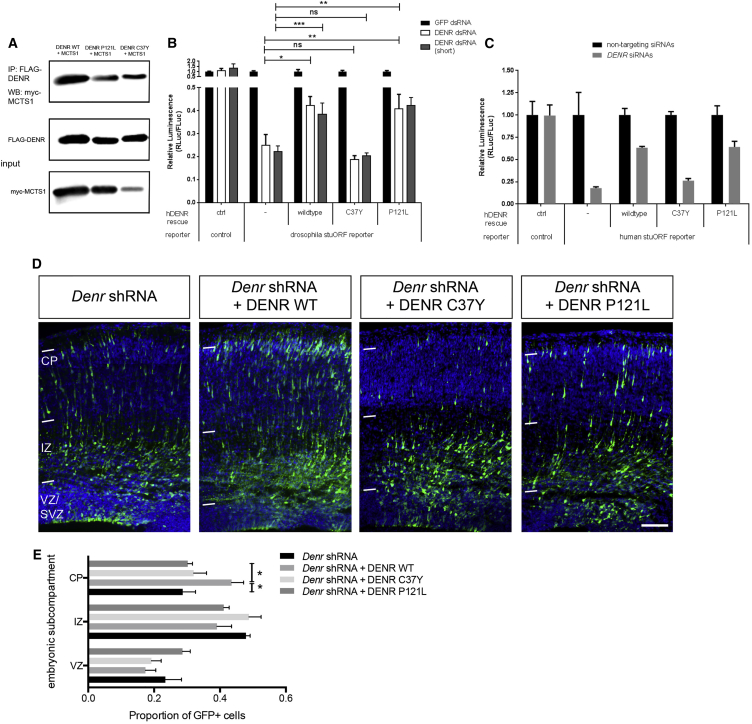
Characterization of DENR and Its Substitution Variants (A) Representative images of co-immunoprecipitation experiments performed in triplicate with lysates of transiently transfected HEK293T cells demonstrate that FLAG-DENR(WT), FLAG-DENR(C37Y), and FLAG-DENR(P121L) immunoprecipitate MYC-MCTS1 in vitro. (B) *Drosophila* S2 cells, treated with dsRNAs to knockdown endogenous DENR, were transfected with a stuORF-containing Renilla luciferase reporter and a control, normalization firefly luciferase reporter, to assay translation downstream of uORFs, together with constructs expressing wild-type or mutant versions of human DENR. In this assay, treatment with human DENR(WT) or DENR(P121L) significantly enhances reporter activity, whereas treatment with DENR(C37Y) does not. (C) DENR and its substitution variants stimulate luciferase reporter activity under the control of a heterologous human stuORF. HeLa cells were treated with non-targeting control siRNA or DENR siRNAs and then re-constituted with constructs expressing DENR(WT), DENR(C37Y), or DENR(P121L) harboring silent mutations, which render them refractory to siRNA-mediated silencing (see Experimental Procedures). Cells were also transfected with an stuORF containing Renilla luciferase reporter and a normalization control firefly reporter to assay translation downstream of stuORFs. DENR(C37Y) is impaired in its ability to stimulate reporter expression, whereas the capacity for DENR(P121L) to stimulate reporter activity is variable, as judged in biological replicates (Figure S5D). (D and E) An in vivo assay to study the capacity for disease-associated DENR variants to restore the defective migration of E14.5-born, GFP-labeled cells treated with *Denr* shRNA and analyzed within the E17.5 cortex. Whereas co-delivery of DENR WT promoted the migration of GFP-labeled, *Denr* shRNA-treated cells into the CP compared to *Denr* shRNA (*Denr* shRNA 0.287 ± 0.039; *Denr* shRNA + DENR WT 0.435 ± 0.036; one-way ANOVA F(6,45) = 4.805; p = 0.0007; Bonferroni multiple comparison ^∗^p < 0.05; n = 3–6) co-treatment with DENR C37Y or with DENR P121L did not augment the defective migration of *Denr* shRNA-treated cells. Graphs plot mean ± SEM. The scale bar (D) represents 50 μm.

DENR was originally characterized as a growth-related protein (Mazan-Mamczarz and Gartenhaus, 2007, Reinert et al., 2006), comprising a SWIB/MDM2 domain predicted to mediate chromatin remodeling and transcriptional activation, as well as an eIF1-like/SUI1 domain known to be important for recognition of the initiation codon and enabling efficient mRNA translation (Kasperaitis et al., 1995, Reinert et al., 2006). We performed amino acid sequence alignment and observed that the C37Y substitution mutation lay within the N-terminal SWIB/MDM2 domain, which is highly conserved from *Drosophila* to humans, whereas the P121L substitution mutation is located adjacent to its C-terminal eIF1/SUI1 domain and does not show evolutionary conservation with *Drosophila* Denr (Figure S5A). In order to understand the potential impact of substitution mutations C37Y and P121L on DENR, we first performed co-immunoprecipitation studies using HEK293T cells transiently transfected with expression constructs encoding FLAG-tagged DENR C37Y and DENR P121L to find that both variants interact with MCTS1 (Figure 5A). In biological repeats of this assay, we observed that the binding capacity of myc-MCTS1 for DENR C37Y and P121L was not significantly different to DENR WT (assessed as the intensity of immunoprecipitated myc-MCTS1 signal relative to input myc-MCTS1 signal; see Figure S5B). However, we also observed a consistent reduction in myc-MCTS1 immunoblotting signal in co-transfection assays with myc-MCTS1 and FLAG-DENR C37Y (see Figure S5C), suggesting in this context that the presence of DENR C37Y could influence myc-MCTS1 expression and, thus, lead to DENR-MCTS1 dysfunction.

Next, we evaluated the mRNA translation function for DENR using two cell-based reporter assays. It was recently reported that one important function for DENR is to regulate mRNA translation during *Drosophila* development through a mechanism involving translation re-initiation after a stuORF sequence (Schleich et al., 2014). We evaluated the capacity of DENR and its substitution variants to reconstitute stuORF-dependent luciferase reporter activity (Schleich et al., 2014) in *Drosophila* S2 cells where endogenous Denr had been knocked down (Schleich et al., 2014). As shown in Figure 5B, whereas knockdown of *Drosophila denr* using two different siRNA reagents (ds DENR and ds DENR short) led to a reduction in stuORF reporter activity, this reduction could be partially but significantly rescued by human DENR WT and P121L, but not by C37Y (Figures 5B and 5F; (4,10) = 88, p < 0.0001 with dsDENR dsRNAs; F(4,10) = 117, p < 0.0001 with short dsDENR dsRNAs; two-way ANOVA with post hoc multiple comparisons testing). In a parallel experiment, we evaluated the potential for DENR and its substitution variants to augment reporter activity under the control of a heterologous human stuORF reporter in HeLa cells. Rescue experiments were performed with expression constructs that were refractory to siRNA-mediated silencing (see Experimental Procedures). Consistent with the findings in our *Drosophila* assays, the C37Y variant was unable to restore reporter activity resulting from siRNA-mediated *DENR* knockdown, whereas both WT and P121L augmented stuORF-mediated reporter activity in this assay (Figure 5C). In biological replicates of this experiment, our results demonstrated that the capacity for P121L to augment luciferase expression under the human stuORF reporter in HeLa cells was variable (Figure S5D), and this variability in reporter activation may be a pathological feature of the P121L substitution variant.

We investigated the possibility that substitution mutations to DENR might disrupt its neuronal functions during cerebral cortical development. We first characterized both FLAG-tagged DENR C37Y and DENR P121L protein expression in E14.5-born CP neurons within the E17.5 cortex by electroporation and observed that their cellular localization is similar to FLAG-DENR (WT; Figure S5E). Next, we electroporated *Denr* shRNA-treated E14.5 cortical cells together with DENR WT, DENR C37Y, and DENR P121L to study the positioning of E14.5-born, GFP-labeled cells within the cortex harvested 3 days later (at E17.5). Our results show that, whereas the migration defect of *Denr* shRNA-treated cells can be corrected with co-delivery of DENR, co-delivery of DENR C37Y or DENR P121L variants failed to correct their defective migration (Figures 5D and 5E). Thus, we find that C37Y and P121L substitution mutations impair the radial migration functions for DENR in vivo.

**Figure 6:**
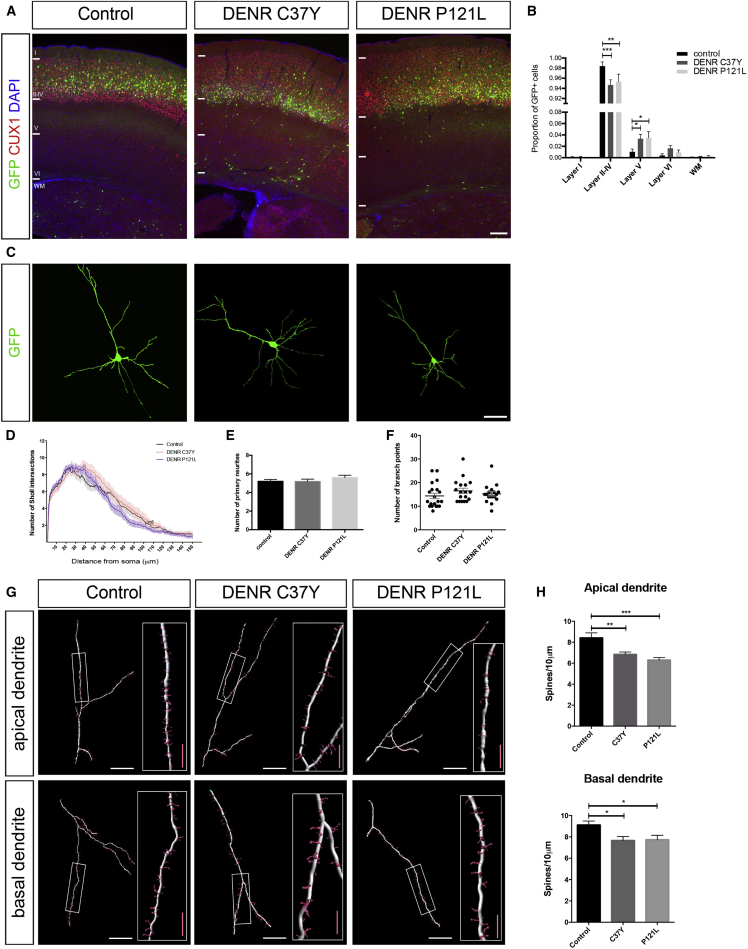
The Presence of DENR C37Y and DENR P121L Disrupts the Long-Term Positioning, Dendritic Branching, and Dendritic Spine Properties of Cortical Projection Neurons (A) In utero electroporation to study the effects of forced expression of DENR C37Y and DENR P121L on the long-term positioning of E14.5-born, GFP-labeled cells within the P17 postnatal cortex, illustrated as representative coronal sections from each. (B) There is a significant interaction between treatment groups and the positioning of GFP+ cells within the P17 cortex (F(8,75) = 3.93; p = 0.0006). Compared to control, there is a significant reduction in the proportion of GFP+ cells in layers II–IV following treatment with DENR C37Y (control 0.984 ± 0.008; DENR C37Y 0.947 ± 0.0103; Dunnett’s multiple comparisons ^∗∗∗^p < 0.005) as well as P121L (control 0.984 ± 0.008; DENR P121L 0.9532 ± 0.0148; Dunnett’s multiple comparisons ^∗∗^p < 0.01), along with a concomitant increase in the proportion of GFP+ cells in layer V in both DENR C37Y- and DENR P121L-treated cells (control 0.010 ± 0.005; DENR C37Y 0.033 ± 0.0077; DENR P121L 0.0348 ± 0.0110; Dunnett’s multiple comparisons ^∗^p < 0.05). (C) Representative images of GFP-labeled, layer II/III cortical projection neurons from each condition. (D) Sholl analysis of dendritic branching complexity reveals alterations to regional dendritic branching complexity in DENR C37Y- and DENR P121L-treated neurons (C37Y: 40–65 μm; P121L: 30–50 μm; as well as Figures S6F and S6G). (E and F) Forced expression of DENR C37Y and DENR P121L did not significantly affect the numbers of primary neurites (two-way ANOVA F(2,52) = 1.041; p = 0.3602; n = 17–20 neurons per condition) or their branch points (two-way ANOVA F(2,52) = 1.022; p = 0.3668; n = 17–20 neurons per condition) when compared with control (GFP-only treatment). (G) Confocal microscopy and image reconstruction of dendritic spines from apical and basal dendrites of GFP-labeled neurons. (H) Following treatment with DENR C37Y or DENR P121L, there is a significant decrease in the density of dendritic spines along apical dendrites (control 8.417 ± 0.4771; DENR C37Y 6.844 ± 0.2217; DENR P121L 6.295 ± 0.2217; one-way ANOVA F(2,49) = 11.62; p < 0.0001; Bonferroni multiple comparisons test ^∗∗^p < 0.01; ^∗∗∗^p < 0.005) as well as basal dendrites (control 9.122 ± 0.3651; DENR C37Y 7.664 ± 0.3635; DENR P121L 7.664 ± 0.3635; one-way ANOVA F(2,52) = 4.759; p = 0.0126; Bonferroni multiple comparisons test ^∗^p < 0.05). Graphs plot mean ± SEM. The scale bars represent (A) 200 μm, (C) 25 μm, (G) 20 μm, and (G insets) 5 μm.

Given that both substitution variants of DENR interact with its in vivo binding partner MCTS1 and thus are predicted to function in a dominant fashion, we studied the effects of their forced expression on the long-term positioning of cortical neurons. In contrast to the lack of an effect of *DENR* WT overexpression on long-term positioning (Figure S3A), we found that forced expression of DENR C37Y or DENR P121L resulted in the defective long-term positioning of E14.5-born cortical neurons within the P17 cortex, observed as a significant decrease in the proportion of GFP-labeled neurons in layers II–IV, and a concomitant increase in the proportion of GFP+ cells residing in layer V (Figures 6A and 6B). The migration profile of neurons overexpressing DENR C37Y and DENR P121L are different to DENR WT overexpression, given that DENR WT overexpression has no significant effect (Figures S6A and S6B, respectively). Given our MRI studies of the patient harboring a *DENR* P121L substitution mutation that revealed features consistent with neuronal migration disorder, including nodular heterotopia (Table S1), we looked for evidence of such a phenotype in our in vivo experiments. We detected the presence of heterotopic clusters of GFP-labeled cells lying adjacent to the ependymal zone in four out of ten (40%) of brains electroporated with DENR P121L, whereas heterotopic clusters of cells were only detected in two out of ten brains electroporated with DENR C37Y (Figures S6C and S6D). This is in contrast to control as well as *Denr* shRNA-treated brains in which heterotopia was detected in only one out of ten (10%) brains. Interestingly, forced expression of human *DENR* in the presence of endogenous mouse *Denr* also led to an increase in heterotopia formation (four out of ten brains [40%]). Immunostaining revealed that clusters of GFP-labeled cells comprised CUX1^+^ neurons as well as CUX1^−^ cells, thereby suggesting that a subset of these cells was correctly specified as upper layer II–IV neurons, but failed to reach their appropriate laminar position. Therefore, our results indicate that, like WT human DENR, forced expression of DENR C37Y and DENR P121L impairs the long-term positioning of cortical neurons and induces the formation of cortical heterotopia, albeit with different potencies.

We investigated the consequences of forced expression of DENR C37Y or DENR P121L on the dendritic arborization and dendritic spine features of P17 cerebral cortical neurons. Our Sholl analyses revealed that forced expression of DENR C37Y or DENR P121L led to an increase in the dendritic complexity of neurons at specific, proximal locations (C37Y: 40–65 μm; P121L: 30–50 μm) relative to the cell soma (Figures 6C, 6D, and S6E–S6G), with no significant changes to the numbers of primary neurites or their branch points (Figures 6E and 6F). However, we found that forced expression of DENR C37Y or DENR P121L did not enhance dendritic complexity to the extent that was observed with forced expression of native DENR (Figures S6E, S6H, and S6I), suggesting that both substitution variants of DENR were disrupted in their capacity to augment dendritic arborization. This finding is consistent with our findings that forced expression of DENR WT, DENR C37Y, and DENR P121L leads to distinct effects on the long-term positioning and the frequency of heterotopia formation.

In addition to changes in dendritic branching, we observed that treatment with DENR C37Y or DENR P121L led to a reduction in dendritic spine densities on apical and basal dendrites (Figures 6G and 6H). However, whereas the presence of DENR C37Y or DENR P121L did not significantly affect the morphology of dendritic spines (Figures S6J and S6K), we found that presence of the P121L variant led to a significant decrease in the volume of mushroom spine heads on apical and basal dendrites (Figures S6L and S6M; summarized in Table S2). Thus, the presence of missense variants of DENR impairs the positioning, terminal arborization, and dendritic spine densities of cerebral cortical neurons.

## Discussion

We have identified important functions for DENR during neurodevelopment. We find that DENR is critical for radial migration within the embryonic cerebral cortex, and this role requires its binding partner MCTS1. In addition, perturbations to *Denr* impair the long-term positioning and terminal differentiation of cortical projection neurons. Furthermore, we have characterized substitution variants of DENR detected in human subjects with neurological disorders and found that the presence of mutated DENR disrupts its functions in mRNA translation in vitro and is detrimental to the development of cerebral cortical neurons in vivo.

In the course of cerebral cortex development, newborn cortical projection neurons undergo a well-documented process of cell migration as they find their appropriate positions within this nascent organ (Kriegstein and Noctor, 2004, Nadarajah et al., 2002, Nadarajah et al., 2003, Noctor et al., 2004). In particular, these neurons undergo a multipolar mode of migration as they transit from the IZ to the cortical plate before completing their migration via radial-glial-guided locomotion as bipolar-shaped neurons (Heng et al., 2010, Noctor et al., 2004). Our studies link DENR-MCTS1 activity to such morphological events during cortical neuron development. Notably, it is recognized that radial migration during cerebral cortex development is sensitive to genetic disruptions that alter the expression levels of critical migration-related factors (Causeret et al., 2009, Heng et al., 2010, LoTurco and Bai, 2006). Hence, our study identifies DENR as a player in this process that acts in a concentration-sensitive manner. Given our finding that DENR is distributed throughout the cell, including in the leading process of CP neurons within the embryonic cortex, we predict that DENR could be important for coordinating the morphology of migrating cells, the orientation of their leading processes for their directional migration, or both. As neurons complete their migration over the course of postnatal neurodevelopment, we find that disruptions to *Denr* impair the long-term positioning of cortical projection neurons. It is noteworthy that forced expression of DENR and its substitution variants disrupted the long-term positioning of cortical projection neurons and induced heterotopia formation in different ways. We conclude that the presence of DENR and its substitution variants leads to distinct consequences for neuronal positioning and suggests that heterotopia formation may be a pathological feature in *DENR* diseased states.

Within the postnatal cerebral cortex, neurons that have completed their migration undergo terminal dendritic branching and form appropriate connections with projection neurons as well as interneurons in a highly organized manner (Harris and Mrsic-Flogel, 2013, Markram et al., 2004). This includes parvalbumin-expressing basket cells targeting the soma and proximal dendrites of cortical neurons to stabilize the activity of local excitatory networks (Harris and Mrsic-Flogel, 2013). We find that knockdown of endogenous *Denr* or overexpression of *DENR* disrupted the dendritic branching of cortical projection neurons proximal (30–50 μm) to their soma and led to a reduction in dendritic spine densities. Thus, our results indicate that perturbations to DENR expression levels could lead to imbalances in excitatory and inhibitory tone, which, in turn, may trigger epileptiform activity, such as was detected in our patient harboring a p.P121L missense mutation. Furthermore, *Denr* gene disruptions significantly altered the shape and volume of mushroom-shaped spine heads (summarized in Table S2). Typically, large spines have proportionally larger synapses and contain a greater diversity of synaptic proteins and organelles, such as smooth endoplasmic reticulum for local synthesis of synaptic proteins (Hering and Sheng, 2001). As it is also known that the size of the spine head is proportional to the area of the post-synaptic density, we predict that DENR is important for the strengthening of synaptic transmission between neurons, with implications for its functions in human brain development and autism spectrum disorder.

In eukaryotes, canonical mRNA translation is carried out on mature cellular mRNAs that bear a 5′ cap structure (m^7^G) and a 3′poly(A) tail (Hinnebusch and Lorsch, 2012). These capped mRNAs are recognized by eIF4F, which functions to recruit the ribosomal 43S complex containing eIF2α to facilitate translation. However, the ribosomal 43S complex can also bind uncapped mRNAs and internal ribosomal entry sites (IRES) of viral transcripts in conjunction with DENR (Skabkin et al., 2010, Skabkin et al., 2013, Zinoviev et al., 2015). DENR facilitates the efficient translation re-initiation of transcripts bearing an stuORF either by release of deacylated tRNA or recruitment of aminoacylated tRNA (Schleich et al., 2014, Skabkin et al., 2010, Skabkin et al., 2013). Another translation factor, ligatin/eIF2D, contains a SWIB/MDM2 domain homologous to DENR in its N-terminal half, as well as an eIF1-like/SUI domain homologous to MCTS1 in its C-terminal half, and has functions in translation that overlap with those of DENR and MCTS1 (Dmitriev et al., 2010, Skabkin et al., 2010, Skabkin et al., 2013, Zinoviev et al., 2015). Whereas it has recently been demonstrated that loss of DENR during *Drosophila* embryo development leads to impaired tissue growth and larval formation (Schleich et al., 2014), reductions in *ligatin* gene dosage were found to exacerbate the defective development of DENR-knockout flies (Schleich et al., 2014), suggesting overlapping functions for DENR and LIGATIN in embryo development. We found that knockdown of *Denr* and *Mcts1*, or both, in the neural cell line Neuro2a did not significantly alter steady-state levels of ligatin (data not shown). Also, *LIGATIN* expression is very weakly detectable in human nervous system tissue compared to *DENR* and *MCTS1* (Figures S1C–S1E), which seems inconsistent with completely redundant roles for DENR/MCTS1 and LIGATIN in the nervous system. Future work will clarify the relative importance for overlapping functions between DENR/MCTS1 and LIGATIN in mammalian neuronal development.

Our study provides evidence for an evolutionary conservation of human DENR function on a *Drosophila* stuORF, and we find that C37Y and P121L substitution mutations disrupt the ability for DENR to facilitate mRNA translation re-initiation through a *Drosophila* stuORF as well as a heterologous human stuORF reporter in different ways. Whereas it will be informative to determine whether both substitutions could disrupt the role for DENR in other aspects of mRNA translation, including ribosome recycling and termination-dependent and -independent re-initiation of viral mRNAs (Skabkin et al., 2010, Zinoviev et al., 2015), our current findings nonetheless provide critical mechanistic insight into the pathological consequences of DENR substitution mutations in mRNA translation and link its altered molecular functions to human neurological disease. As such, our studies provide an important context for DENR-MCTS1 functions within the mammalian nervous system and suggest that neural cells require activity of DENR-MCTS1 for regulating mRNA translation so as to coordinate cell migration and terminal neurodifferentiation during cerebral cortex development.

It is noteworthy that a C37Y substitution variant was detected in a patient diagnosed with autism, whereas a P121L mutation was detected in a patient diagnosed with epilepsy as well as Asperger syndrome, an autism spectrum disorder. Given the compelling documentary evidence for abnormal cortical organization (Stoner et al., 2014) and defective neuronal positioning (Wegiel et al., 2010) in children diagnosed with autism, our findings are consistent with the notion that certain clinical presentations of autism spectrum disorder could be delineated on the basis of candidate gene mutations, including to *DENR*, which disrupt core signaling pathways for neural circuit organization and synaptic function (Liu et al., 2014, Neale et al., 2012). Our future investigations will clarify the nature of DENR missense substitutions in human neuronal development and define their precise roles in the pathogenesis of pediatric neurological disease.

## Experimental Procedures

### Exome Sequencing

The collection and sequencing of DNA from the index patient with structural brain abnormalities is authorized under two separate ethical frameworks administered by the Wales Research Ethics Committee (08/MRE09/55 and 12/WA/0001) of the UK. Whole-exome sequencing was performed on DNAs extracted from patient and parental blood samples. Libraries were prepared using standard procedures, captured using the SureSelect Human all exon kit (Agilent Technologies), and then sequenced using Illumina technology. The 51-bp paired-end reads were mapped using Stampy (Lunter and Goodson, 2011), and duplicate PCR reads were removed using Picard (http://picard.sourceforge.net/). Single-nucleotide variants were called using Platypus (Rimmer et al., 2014).

### Animals

C57BL/6J wild-type mice were bred, housed, and time mated according to standard operating procedures approved by the Animal Ethics Committee at Monash University (MARP/2012/069) and UWA (AE201) and compliant with guidelines stipulated by the National Health and Medical Research Council of Australia.

### Expression Constructs

Expression constructs were prepared according to standard molecular cloning procedures. N-terminal MYC-tagged MCTS1 cDNA was cloned into pCIG2; human *DENR* and *DENR* substitution mutations c.G110A and c.C362T were cloned into pCIG-Flag expression vector, as well as with a pCIG-Flag vector in which the GFP cassette was excised by restriction digestion and subsequent cloning. *Denr* oligonucleotides with appropriate hairpin sequences targeting mouse *Denr* (1: 5′-CCAAGTTAGATGCGGATTA; 2: 5′-CCACAGAAGGTCACGATAG) were cloned into the pSilencer-EGFP expression vector, and a non-targeting scrambled control pSilencer-EGFP (Bron et al., 2007) was used as the control in all RNAi experiments. All expression constructs used in vivo were prepared using QIAGEN DNA purification products with endofree reagents, according to manufacturers’ instructions, and eluted in water. All products were sequence verified. ON-TARGET plus SMARTpool siRNAs targeting mouse *Denr* and *Mcts1* were purchased from Dharmacon and reconstituted in water for use at 5 μM. The efficacy of knockdown by siRNAs and shRNAs were confirmed by Lipofectamine transfection with the neuroblastoma Neuro-2A cell line, lysates of which were harvested for western blotting or qRT-PCR 48 hr later.

Luciferase reporter constructs for testing in *Drosophila* S2 cells were as follows: a Firefly reporter pGL3:hsp70>>firefly luciferase, containing the 5′ UTR of *Drosophila* gene CG4637 (cloned via PstI/NcoI), served as a normalization control. pGL3:hsp70>>Renilla luciferase containing the same 5′ UTR, with or without a synthetic stuORF, served as the experimental readout. Luciferase reporter constructs for testing in HeLa cells were constructed via a multistep cloning procedure, resulting in two plasmids, each bearing both a Renilla luciferase gene as well as a normalization control firefly luciferase gene, with the difference that one of the two plasmids contains a stuORF upstreamof the Renilla luciferase (the “stuORF reporter”) whereas the control reporter plasmid does not. The plasmids have the following features in sequential order: pGL3Promotor (Promega) backbone; a cytomegalovirus (CMV) promotor; 5′ UTR of *Drosophila* gene CG43674; Firefly luciferase reporter ORF; SV40 polyA; CMV promotor; 5′ UTR of CG43674 (without stuORF in the control reporter or bearing a synthetic stuORF in the stuORF reporter); Renilla luciferase reporter ORF; and SV40 polyA. Human DENR ORF was amplified by PCR from HeLa cDNA with EcoRI and NotI overhangs. The PCR product was cloned into the TOPO TA vector (Invitrogen), fully sequenced, and subsequently subcloned into the EcoRI-NotI sites of pCDNA3. To render the hDENR ORF insensitive to knockdown with hDENR siRNA (Dharmacon siGENOME siRNAi D-012614-02), the siRNA target site was mutated by point mutagenesis to introduce silent mutations using oligos OSS388 and OSS389, yielding the “wild-type” rescue construct. The C37Y or P212L mutations were then introduced by site-directed mutagenesis. For expressing in *Drosophila* S2 cells, wild-type and C37Y or P212L mutant hDENR ORFs were cloned into pMT via EcoRI and NotI sites. For all constructs utilized in this study, full sequences are available upon request.

### Cell Culture, Western Blotting, and Co-immunoprecipitation

Cell culture for immunoprecipitation assays and western blotting was performed as previously described (Breuss et al., 2012, Gladwyn-Ng et al., 2015). Antibodies used in this study (listed by species) include mouse anti-immunoglobulin G (IgG) (Millipore) anti-β-actin (A5441; Sigma-Aldrich), anti-FLAG (F1804; Sigma-Aldrich), anti-MYC (Sigma-Aldrich), anti-Tuj1 (MMS-435P; Covance), anti-GFAP (Millipore), anti-DENR (Santa Cruz Biotechnology); rabbit anti-DENR (Novus Biologicals), anti-MCTS1 (Novus), anti-FLAG (Cell Signaling Technology), anti-CDP (sc-6327, Cux1; Santa Cruz), anti-activated caspase-3 (R&D Systems), anti-Myc (Abcam); goat anti-MCTS1 (Santa Cruz); and chicken anti-GFP (Ab13970; Abcam). Preimmunized goat serum (Sigma) was used for immunoprecipitation experiments as a control. Cell nuclei were visualized with DAPI.

For experiments with *Drosophila* S2 cells, double stranded RNA (dsRNA)-targeting dDENR was added to medium at a concentration of 12 μg/ml and incubated for 3 days. Following this, luciferase reporter constructs and hDENR rescue constructs where co-transfected with Effectene (QIAGEN) according to manufacturer’s instructions. On the 4^th^ day, medium was changed to fresh serum-free media (SFM) containing additionally 0.175 mg/ml of copper sulfate for induction of “rescue” protein expression and luciferase assays were performed the following day. When conducting luciferase reporter assays with HeLa cells, 96-well microtiter plates were seeded at 12,000 cells/well. Knockdown was achieved with a mix of three different siRNAs from Dharmacon targeting hDENR (D012614-19 and D-012614-20 targeting the 3′ UTR of endogenous hDENR plus D-012614-02 targeting the coding sequence, which was mutated in the rescue constructs mentioned above), or negative control siRNA (Ambion) at a final concentration of 5 pmol/well was performed with RNAiMax (Life Technologies) and incubated for 2 days. Following this, cells were transfected with pCDNA3 rescue constructs (wild-type, C37Y, or P121L) and luciferase reporter plasmids and incubated another day before the dual luciferase assay was performed. Where relevant, signals between conditions were evaluated using Student’s t tests or one-way or two-way ANOVA analyses followed by an appropriate post hoc t test corrected for multiple testing.

### qRT-PCR, Western Blotting, and Immunoprecipitation

For qRT-PCR, total mRNA was extracted from mouse brain tissues at five different time points, E11.5, E14.5, P0, P10, and young adult (P30) was used for reverse transcription. Quantitative real-time PCR employed exon-spanning primers, *Denr* (forward: 5′-AAAAATTCTCGTGCGGTGCC; reverse: 5′-CATCCGCCTCTGGCCACTTT) and *Mcts1* (forward: 5′-CTTGCCACATCAGCAGGTTG; reverse: 5′-AGCTTAGCTCCGGGAGAAGT), with *Pgk1* (forward: 5′-AAACTCAGCCATGTGAGCACT; reverse: 5′-ACTTAGGAGCACAGGAACCAAA) as an internal control gene. Each qPCR reaction consisted of 10 ng/μl template cDNA, 5 μM target gene primers, or 10 μM housekeeping gene primers. The levels of *Denr* and *Mcts1* were expressed relative to *Pgk1*, and signals between conditions were evaluated using Student’s t tests.

Immunoprecipitation and western blotting were carried out as follows. Briefly, cultured cells and brain tissue were lysed using lysis buffer (20 mM Tris [pH 7.5], 150 mM NaCl, 1% IGEL-PAL, 0.1% SDS, and protease inhibitor), whereas brain tissue for immunoprecipitation experiments was lysed using Promega Cell Culture Lysis 5× Reagent (cat. no. E153). Brains were homogenized and protein concentration measured using Bradford reagent (Bio-Rad). For immunoprecipitation, 1 mg protein was incubated with 1.0–1.5 μg antibody overnight at 4°C with rotation, followed by incubation with protein A Sepharose beads for 2 hr at 4°C. Immunoprecipitated proteins were eluted with 50 mM glycine. Protein samples were analyzed on a 10% SDS gel, transferred to nitrocellulose membrane, and incubated overnight with primary antibodies. Membranes were incubated with anti-rabbit LI-COR Biosciences IRDye 680 LT or anti-mouse LI-COR Biosciences IRDye 800 secondary antibodies for 1 hr before analysis using the Odyssey imaging system (LI-COR Biosciences; 9201-02).

### In Utero Electroporation

In utero electroporation was performed according to the methodology developed by Tabata and Nakajima (2003), with modifications we have described previously (Haas et al., 2013, Ngo et al., 2014). Briefly, time-mated pregnant (E14.5) C57BL/6J female mice were anesthetized with sodium pentobarbitone and incisions made in the skin and peritoneum. The uterus was exposed from the abdomen, and the lateral ventricle of embryos was injected with a solution containing 1 μg/μl of each species of DNA expression plasmid, 5 μM siRNA (when indicated), and Fast Green tracer dye. The quantities of expression plasmid (1 μg/μl), shRNA vector (1 μg/μl), and siRNAs (5 μM siRNA) for injection were balanced for all electroporation experiments to enable comparisons between conditions in our data. Following electroporation with a square-pulse generator, the uterine horns were returned to the abdominal cavity and the peritoneal and skin incisions sutured. Electroporated brains were harvested after 3 days (E17.5) or at P17. Where relevant, migration profiles between conditions were evaluated through two-way ANOVA analyses followed by an appropriate post hoc t test corrected for multiple testing.

### Tissue Collection, Fixation, and Immunohistochemistry

At E17.5, brains were dissected and fixed overnight in 4% paraformaldehyde (PFA), whereas P17 mice were anesthetized with sodium pentobarbitone and transcardially perfused with PBS followed by 4% PFA and then post-fixed overnight in 4% PFA. Brains were cryoprotected in 20% sucrose and then cryostat sectioned at 16 μm (E17.5) or 40 μm (P17) thickness. P17 sections underwent free-floating immunohistochemistry, whereas E17.5 immunohistochemistry was performed on slides, using standard protocols (Heng et al., 2008, Ngo et al., 2014). Sodium citrate (0.01 M) antigen retrieval was used in some cases to enhance immunolabeling. Species-appropriate Alexa Fluor (Molecular Probes) secondary antibodies were used for immunofluorescence detection. For E17.5 experiments, images were captured using an Olympus microscope equipped with a charge-coupled device (CCD) camera (SPOT). Subdivisions of the embryonic cortex (VZ, IZ, and CP) were identified based on cell density visualized by DAPI staining. Cell counting was performed blind to the condition on representative fields of sections of electroporated brains using ImageJ (1.47 m) software.

For in utero studies that focus on GFP-labeled neurons within the P17 cortex, electroporations were performed with injection mix, which comprised equal quantities of expression vector and shRNA vector to enable comparisons between conditions. That is, 1 μg/μl of pCIG construct was co-delivered with 1 μg/μl of pSil-Caggs shRNA vector (control, pCIG + psil-Caggs-scr; shRNA, pCIG + pSil-Caggs-*Denr* shRNA1; DENR, pCIG-F-DENR + pSil-Caggs-scr; C37Y, pCIG-F-DENR(C37Y) + pSil-Caggs-scr; P121L, pCIG-F-DENR(P121L) + pSil-Caggs-scr). GFP-labeled P17 neurons were captured as 3D confocal images of 40-μm-thick mouse brain sections and digitally reconstructed using Filament Tracer (Imaris 7.6.2; Bitplane). This analysis was conducted on GFP-labeled cortical projection neurons residing within layers II/III of the somatosensory cortex of successfully electroporated P17 brains. For cell morphology analysis, neurons were captured at 40× magnification, whereas for dendritic spine studies, neurites were imaged at 60× magnification, with 1 μm z-stack step size. Both rendering of neuronal cell morphology as well as the identification of dendritic spines were performed manually, whereas the classification of spine types was automatically calculated based on pre-defined parameters, as described recently (Ngo et al., 2014). Representative images of projection neurons identified from each condition were prepared with Photoshop using non-linear image manipulation. Imaris Software reported results from statistical tests related to the analyses of neuronal morphology and dendritic spines properties.

## Author Contributions

J.I.-T.H. conceived this study with M.A.H. and L.N. M.A.H., S.S., and H.K.V. performed molecular cloning and immunoprecipitation assays, whereas in utero electroporation experiments were performed by M.A.H., Z.Q., I.E.G.-N., and H.D.C. Protein biochemistry studies and gene expression assays were performed together with L.N., S.S.L., I.E.G.-N., H.K.V., A.C.-A., and K.E.D. S.S. and A.A.T. performed luciferase reporter assays, whereas microscopy analysis was performed by M.A.H. and L.N. A.T.P., H.S., J.C.T., U.K., and D.A.K. collected and collated the clinical data and carried out genetic screening and sequencing. M.A.H. and L.N. prepared the figures with J.I.-T.H. J.I.-T.H. wrote the manuscript with M.A.H. and L.N., and all authors provided comments.

## Supplemental Information

Document S1. Figures S1–S6 and Tables S1 and S2

Document S2. Article plus Supplemental Information
