# Supplementary material for: De Novo Mutations in DENR Disrupt Neuronal Development and Link Congenital Neurological Disorders to Faulty mRNA Translation Re-initiation
Source: Cell Rep. 2016 May 26;15(10):2251–65. doi: 10.1016/j.celrep.2016.04.090 (PMC4906373; doi:10.1016/j.celrep.2016.04.090)
Supplement: Document S1. Figures S1–S6 and Tables S1 and S2 [file mmc1.pdf]

Cell Reports, Volume 15

Supplemental Information

De Novo Mutations in DENR Disrupt Neuronal Development and Link Congenital Neurological Disorders to Faulty mRNA Translation Re-initiation

Matilda A. Haas, Linh Ngo, Shan Shan Li, Sibylle Schleich, Zhengdong Qu, Hannah K. Vanyai, Hayley D. Cullen, Aida Cardona-Alberich, Ivan E. Gladwyn-Ng, Alistair T. Pagnamenta, Jenny C. Taylor, Helen Stewart, Usha Kini, Kent E. Duncan, Aurelio A. Teleman, David A. Keays, and Julian I.-T. Heng

Supplemental Figure S1 related to Figure 1

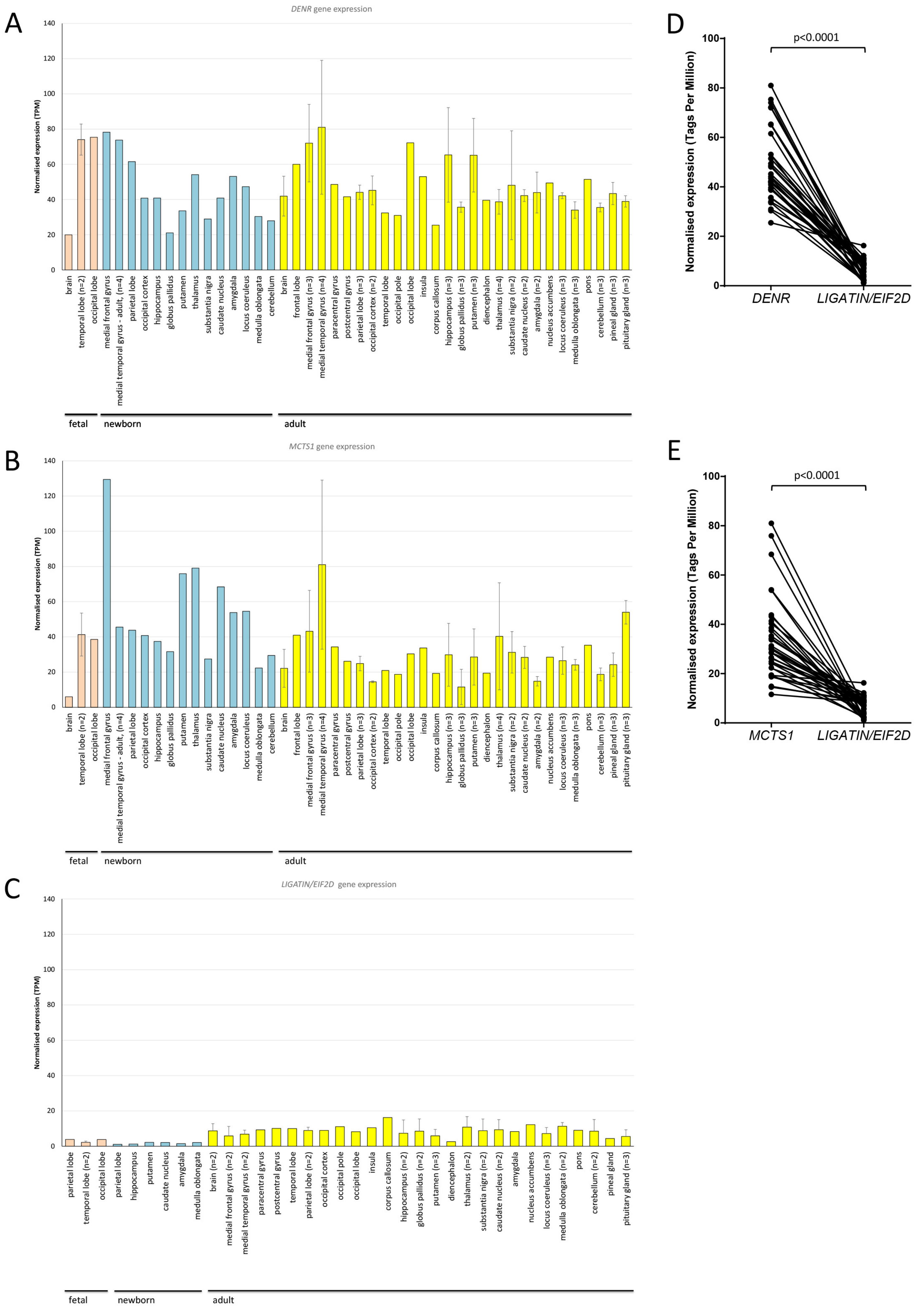

Supplemental Figure S2 related to Figure 2

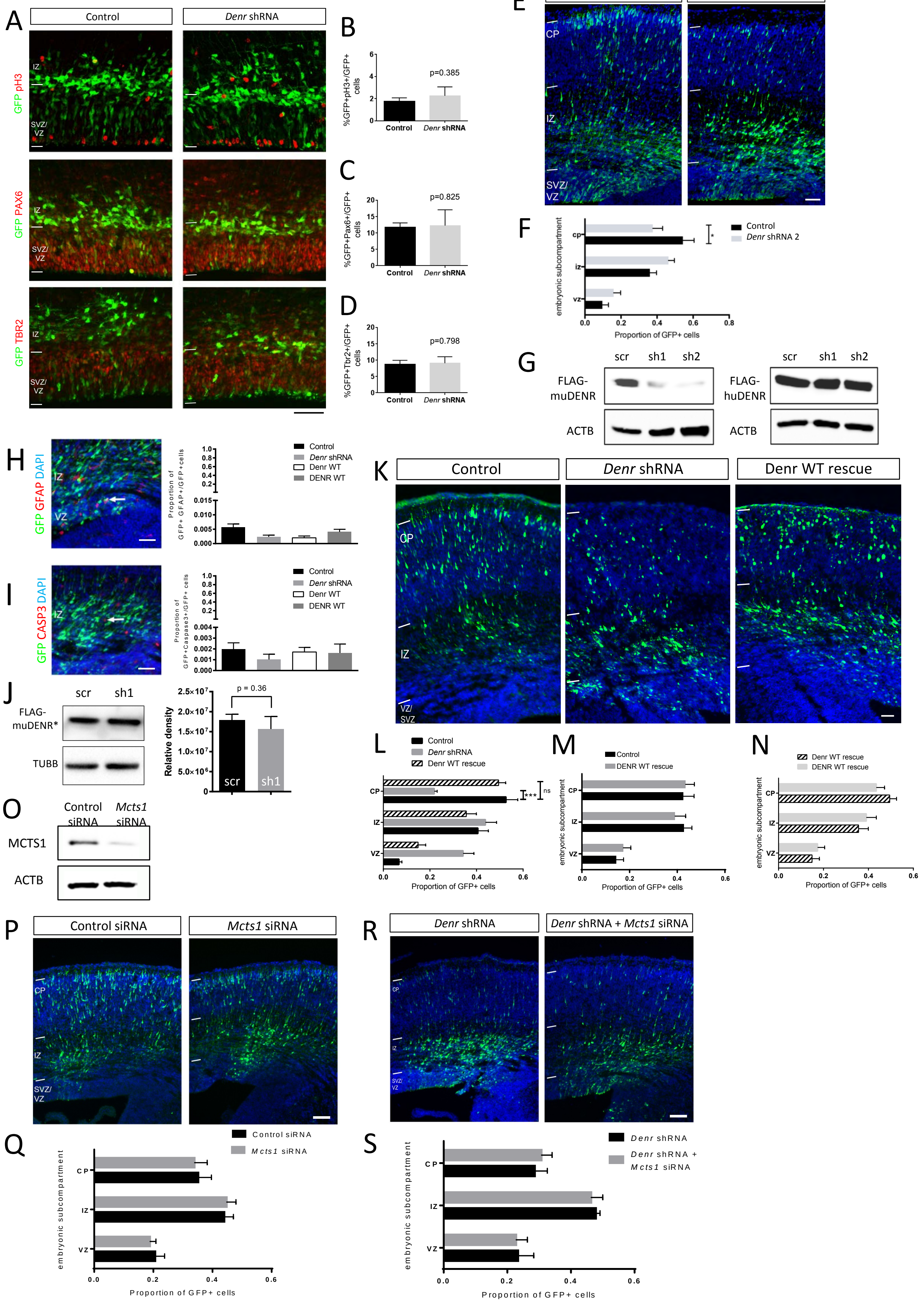

Supplemental Figure S3 related to Figure 3

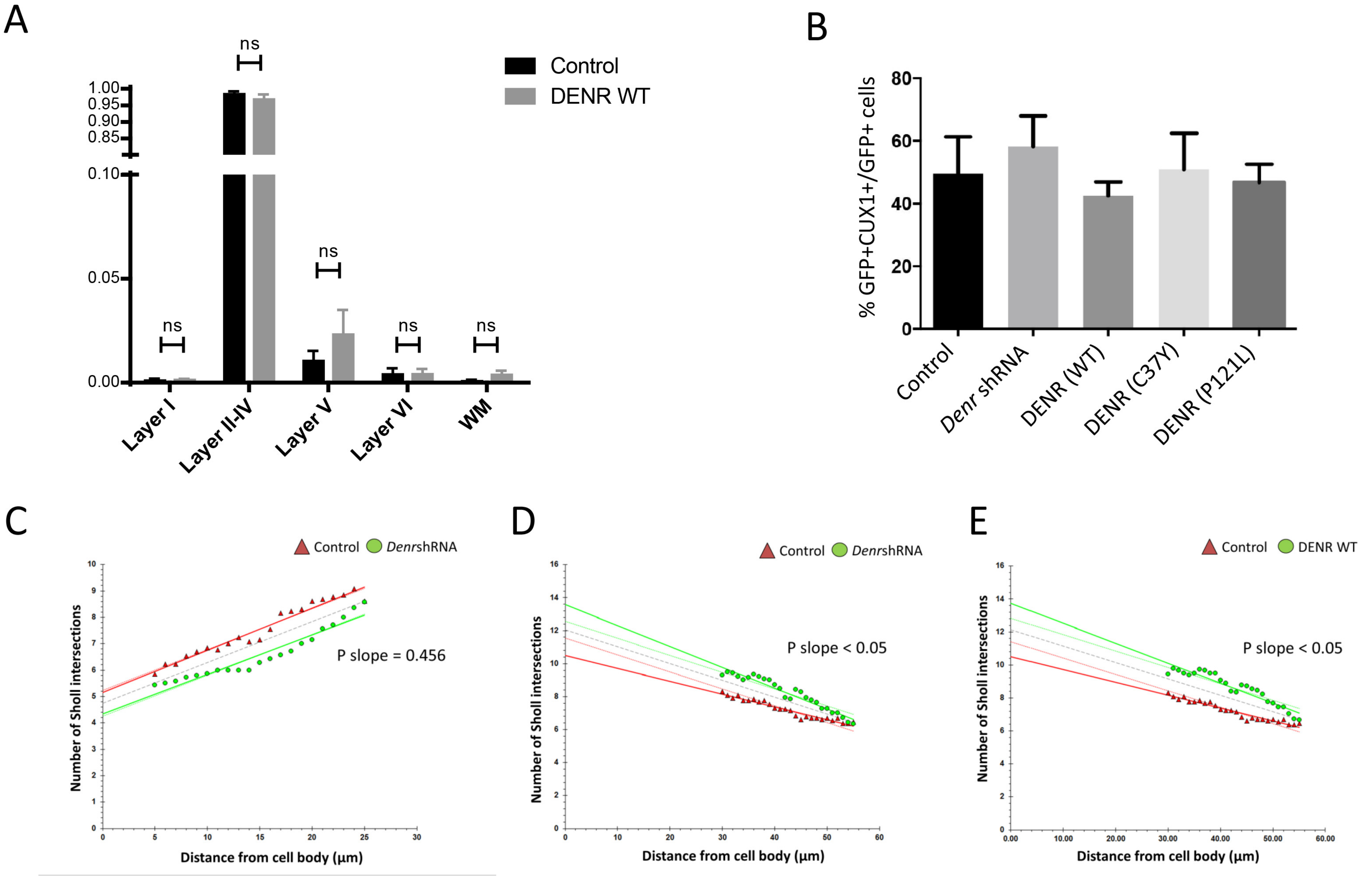

Supplemental Figure S4 related to Figure 4

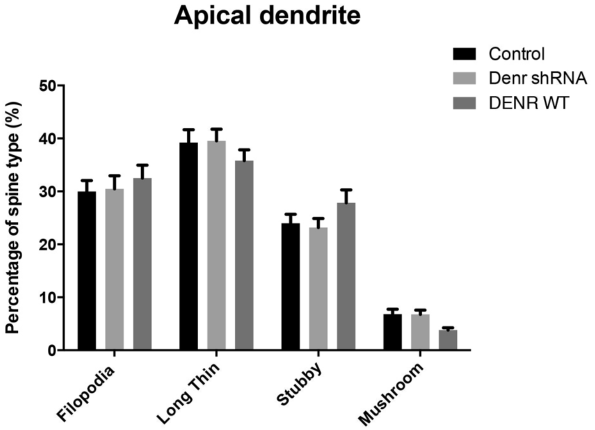

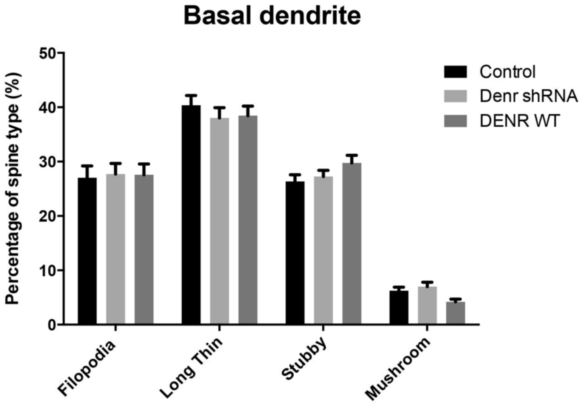

Supplemental Figure S5 related to Figure 5

A

```
Homo sapiens                    ---MAADISESSGADCKGDPRNSAKLDADYPLRVLYCGVCSLPTEYCEYMPDVAKCRQWL 57
Mus musculus                    ---MATDISESSGADCKGDTKNSAKLDADYPLRVLYCGVCSLPTEYCEYMPDVAKCRQWL 57
Xenopus Laevis                  MASIAIENAEPVPSDCKGDLKIS-KFDGDLPLKVLYCGVCSLPTEYCEYMPDVAKCRQWL 59
Danio rerio                     --MATTENAESGSPENKVDRADP---DAKYPLKVLYCGVCSLPAEYCEYMPEPAKCKQWL 55
Drosophila melanogaster         --MTNEDVGTNSVADRLQLGPRE---GVTYPIQMKYCGHCTMPIEYCEYYPEYEKCKEWL 55
Caenorhabditis elegans          --MSD---GETAVIQSLAPGPID---GVSYPLKMVYCGQCSMPPEYCDYSGQTDVCRAWA 52
                                        .     :           .   *::: *** *::* ***:*  :   *: *

Homo sapiens                    EKNFPNEFAKLTVENSPKQEA----GISEGQG--------TAGEEEEKKKQKRGGRGQIK 105
Mus musculus                    EKNFPNEFAKLTVENSPKQET----GITEGQG--------PVGEEEEKKKQKRGGRGQIK 105
Xenopus laevis                  EKNFPDEFSKLTLGISPKQET----GTVEGQA--------TSGEEEEKKKQKRGGRGQIK 107
Danio rerio                     EKNFPDVFAKLTLGTAPKQESKGGGGGEDGGGGRGRGEAPPAGEEEEKKKQKRGGRGQIK 115
Drosophila melanogaster         ELHMPDDFERLKIEEEAAAADG---------------------TDDDKKRQKRGGKGLLR 94
Caenorhabditis elegans          TQNAPELLEGLEISDEPAADG------------------------DEKKKQKRGGKGSKT 88
                                  : *: :  * :   .                            ::**:*****:*

Homo sapiens                    ------------QKKKTVPQKVTIAKIPRAKKKYVTRVCGLATFEIDLKEAQRFFAQKFS 153
Mus musculus                    ------------QKKKTVPQKVTIAKIPRAKKKYVTRVCGLATFEIDLKEAQRFFAQKFS 153
Xenopus laevis                  ------------QKKKTVPQKVTIAKIPRAKKKYVTRVCGLATFEIELKDAQRFFAQKFS 155
Danio rerio                     ------------QKKKTVPQKVTIAKIPRAKKKYVTRVCGLATFDIELKEAQRFFAQKFS 163
Drosophila melanogaster         -----------VKKKEDVPKRICVSRAARGKKKSVTVVTGLSTFDIDLKVAAKFFGTKFA 143
Caenorhabditis elegans          GAAAAQAAASGGKKKGGGPQKVTLQREPRGKK-SVTVIKGLATFDIDLKVASKLFAQKFA 147
                                            :**   *::: : : .*.**  ** : **:**:*:** * ::*. **:

Homo sapiens                    CGASVTGEDEIIIQGDFTDDIIDVIQEKWPEVDDDSIEDLGEVKK- 198
Mus musculus                    CGASVTGEDEIIIQGDFTDDIIDVIQEKWPEVDDDSIEDLGEVKK- 198
Xenopus laevis                  CGASVTGEDEIIIQGDFTDDIIDVIQEKWPEVDDDSIEDLGEVKK- 200
Danio rerio                     CGASVTAEDEIIIQGDFTDDIIDVIQEKWPEVDDDSIDDLGEVKK- 208
Drosophila melanogaster         CGSSVTGDDEIVIQGDVKDDLFDVIPEKWAEIDEDVIEDLGDQKRT 189
Caenorhabditis elegans          CGSSVTGADEIVIQGDVKDDLLDLIPEKWKQVTDEQIDDLGDKKR- 192
                                **:***. ***:****..**::*:* *** :: :: *:***: *:
```

B

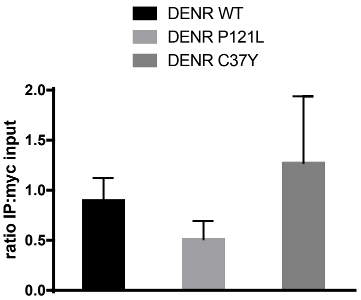

C

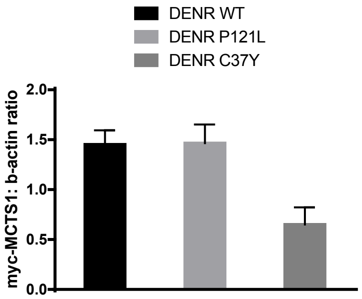

D

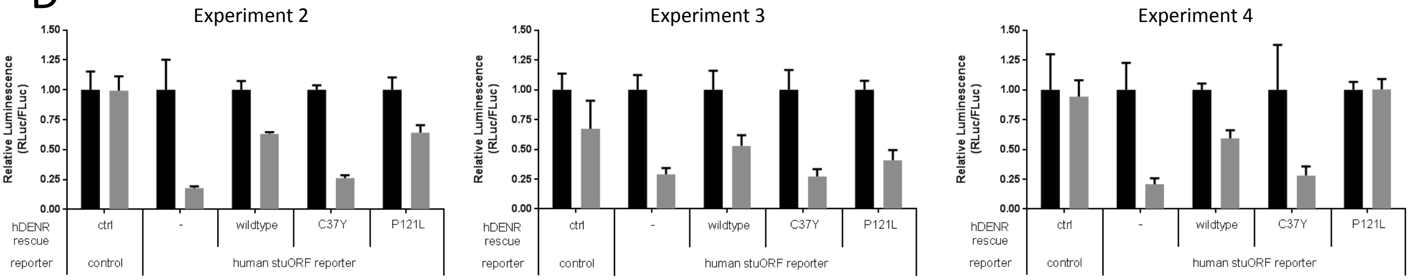

E

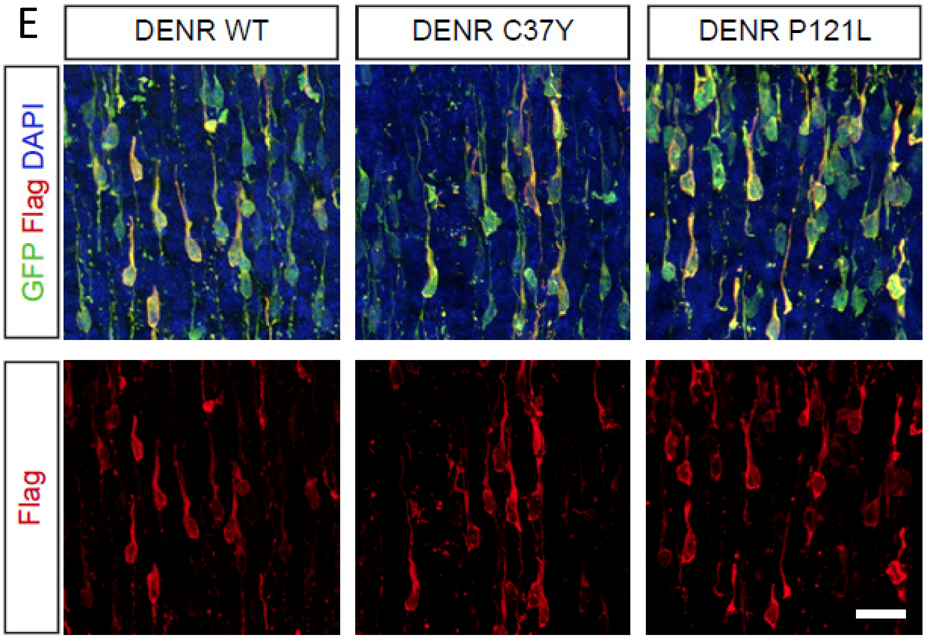

Supplemental Figure S6 related to Figure 6

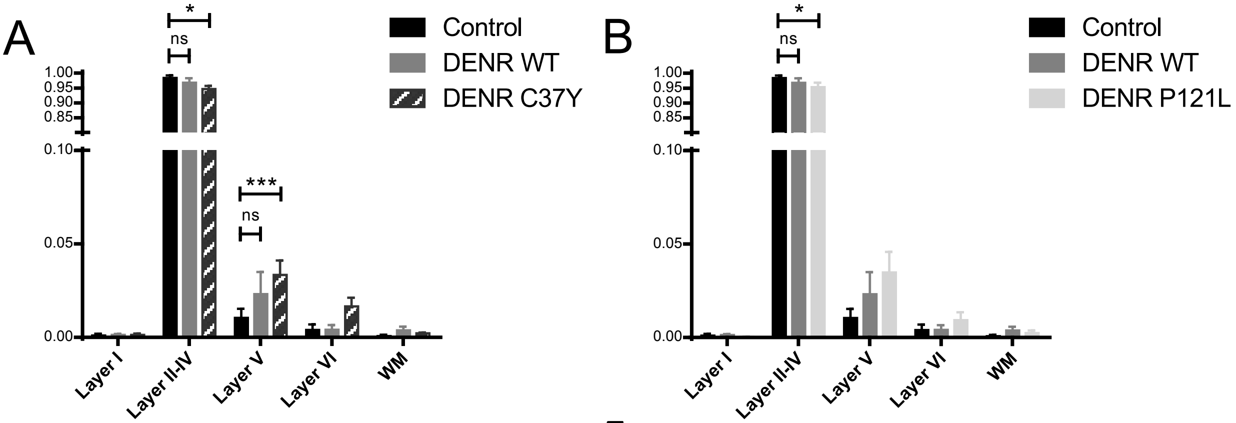

C

Condition	Number of electroporated brains comprising heterotopic clusters	Cellular composition of heterotopic clusters	
		$GFP^{+}CUX1^{-}$ cells in clusters only	mixed
**Control**	1 out of 10		✓
***Denr* shRNA**	1 out of 10	✓	
**DENR(WT)**	4 out of 10	✓	✓✓✓
**DENR(C37Y)**	2 out of 10		✓✓
**DENR(P121L)**	4 out of 10	✓	✓✓✓

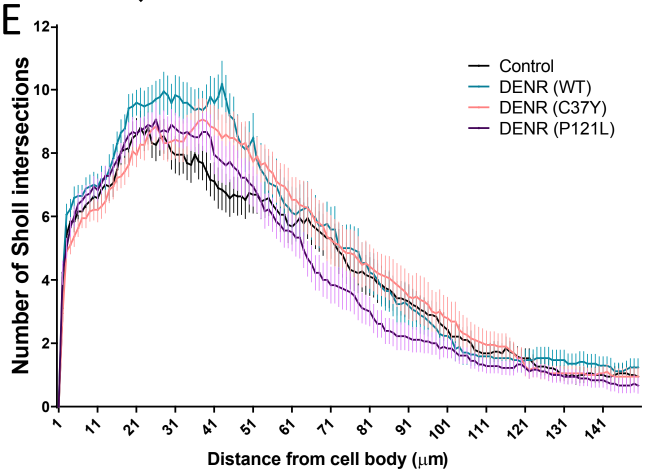

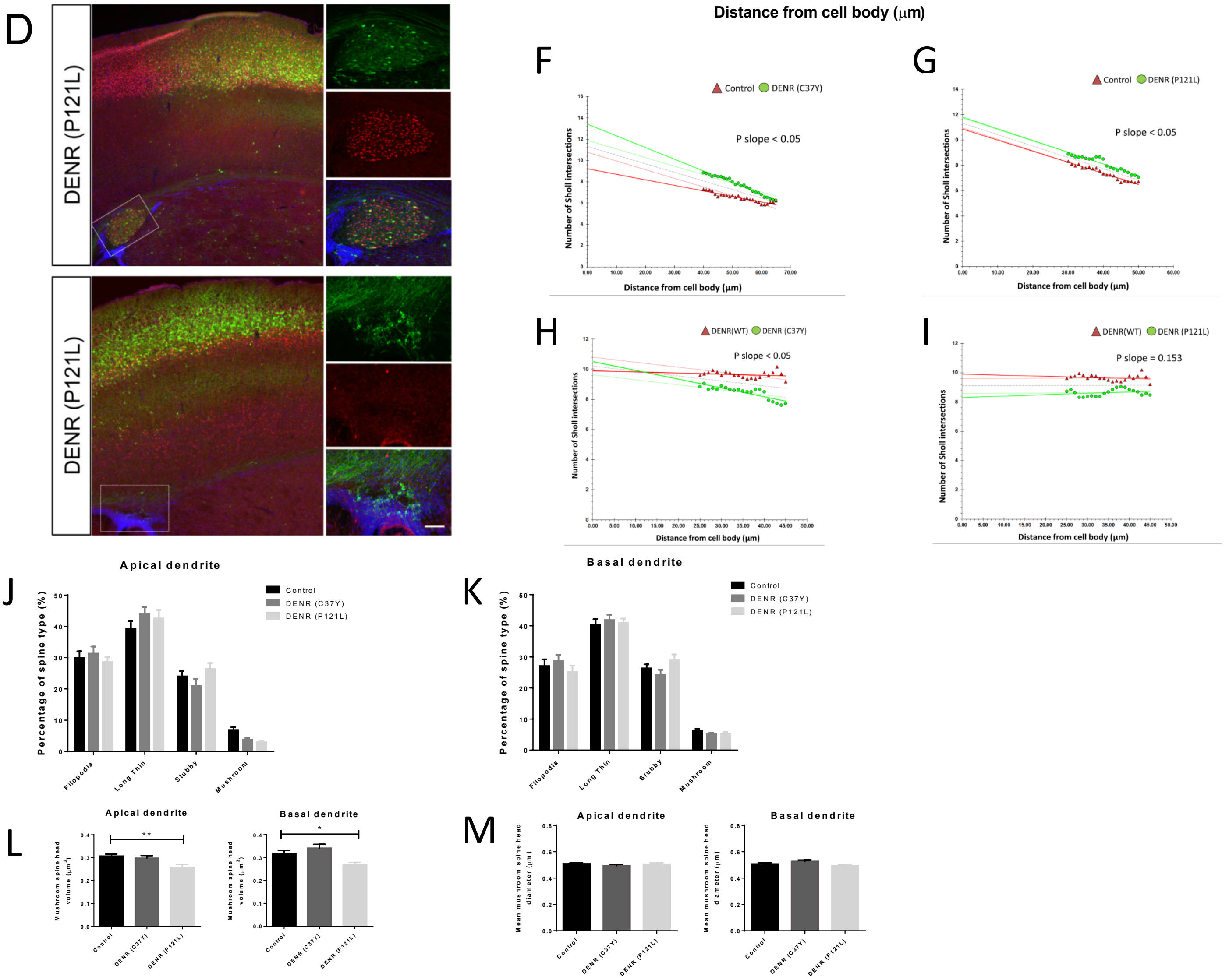

LEGENDS TO SUPPLEMENTARY FIGURES

Figure S1 related to Figure 1. (A to B) A survey of *DENR* and *MCTS1* expression in human brain tissue samples from human fetal (pink bars), newborn (light blue) and adult (yellow bars) using Capped Analysis of Gene Expression (CAGE) in FANTOM5 (Consortium et al., 2014). (C) An expression profile for the related factor *LIGATIN* is also provided. Quantitative data was normalized across libraries and expressed as Tags Per Million (TPM) mapped reads in a given CAGE library. Where multiple samples were available from a given tissue, data is plotted as an average $\pm$ standard deviation. As a guide, expression levels at 10 TPM reflect approximately 3 copies of a given transcript in a cell (Consortium et al., 2014). (D) The relative expression level of *DENR* across brain tissues was significantly higher than *LIGATIN* ($p<0.0001$, n=34 brain tissue libraries where expression data (TPM>0) was available for both genes). (E) The relative expression level of *MCTS1* across brain tissues was also significantly higher than *LIGATIN* ($p<0.0001$, n=34 brain tissue libraries where expression data (TPM>0) was available for both genes).

Figure S2 related to Figure 2. Evaluating *Denr* shRNA effects on cortical neuroprogenitors, as well as the effects of *Denr* shRNA and *Mcts1* siRNA treatment on cell migration within the embryonic mouse cerebral cortex.

(A) *In utero* electroporation was performed on E14.5 embryos to investigate the effects of *Denr* shRNA treatment on cortical progenitors within the E16.5 cortex. The proportion GFP-labelled cells undergoing cell division, indicated by co-expression of the M-phase mitosis marker phosphorylated histone H3 (pH3), was not significantly different (B). Co-labelling for the neuroprogenitor marker Pax6, as well as the basal progenitor marker Tbr2 in parallel experiments demonstrate that treatment with *Denr*

shRNAs did not significantly affect the co-expression of these markers in labelled cells (C and D, respectively, n=6 control and 5 *Denr* shRNA-treated brains per condition). (E) *In utero* electroporation studies of an additional, *Denr* shRNA 2 introduced into cells of the E14.5 embryonic cortex and analysed three days later at E17.5. (F) Treatment with *Denr* shRNA 2 leads to a significant reduction in the proportion of GFP+ cells within the CP (Control 0.543±0.062, shRNA 2 0.377±0.054, 2-Way ANOVA F(4,54)=3.618, P=0.0110, with Bonferroni multiple comparisons *p<0.05, n=6-8 per condition). (G) Immunoblotting studies with *Denr* shRNA 1 and shRNA 2 showing knock down of FLAG-tagged mouse DENR (FLAG-muDENR) signal in lysates of transiently transfected Neuro2A cells. Parallel experiments show that both *Denr* shRNAs do not suppress FLAG-humanDENR (FLAG-huDENR) immunoblotting signal. (H to I) There is no significant difference in the proportion of cells which express the glial cell marker, GFAP (1-way ANOVA F(3,24)=2.7, P=0.0654) nor the pro-apoptotic marker, activated Caspase-3, between the conditions indicated (1-Way ANOVA F(3, 22)=2.7, P=0.681). (J) Evaluation of an expression construct comprising mouse wildtype *Denr* cDNA clone (designated as muDenr*) in pCIG-F vector which is refractory to *Denr* shRNA-mediated knockdown, as demonstrated a Western blotting assay with protein lysate from Neuro2a cells transiently transfected with a non-targeting shRNA (scr) or *Denr* shRNA 1 (sh1). In biological triplicate experiments, there is no significant difference in the amount of immunodetectable FLAG-muDenr* between shRNA treatments. (K to L) The defective migration of *Denr* shRNA-treated cells can be restored by co-delivery of muDenr* (denoted as “Denr WT rescue”). As shown, while *Denr* shRNA treatment leads to a significant impairment in GFP+ cells within the CP, this defect can be rescued by mouse Denr to levels which are not significantly different to control

profile within the CP (Control 0.526±0.056, *Denr* shRNA 0.281±0.014, Denr WT rescue 0.494±0.035, n=4-5 brains per condition, 2-Way ANOVA F(4,30)=16, P<0.0001, with Bonferroni multiple comparisons). (M) The migration profile of GFP-labelled cells treated with *Denr* shRNA together with DENR WT was not significantly different to Control whereby a GFP-only vector was co-delivered with a non-targeting shRNA vector (2-way ANOVA F(2,24)=0.4278, P=0.6568). (N) The migration profile of mouse Denr WT rescue cells and human DENR WT rescue cells was not significantly different (2-way ANOVA F(2,21)=0.95, P=0.4036). (O) Western blotting analysis with transiently transfected Neuro2a cells demonstrates that *Mcts1* siRNAs suppress Mcts1 immunoblotted protein signal. (P and Q) Treatment with *Mcts1* siRNAs did not significantly perturb migration (2-way ANOVA F(2,33)=0.07616, P=0.7930). (R and S) The defective migration of *Denr* shRNA-treated cells was not exacerbated upon co-delivery of *Mcts1* siRNAs (2-way ANOVA F(2,21)=0.1080, P=0.8982). Values represent mean ± SEM. Scale bar = (A, E, K, P, R), 50μm; (H and I), 50μm (H and I insets) 15μm.

Figure S3 related to Figure 3. The effects of *Denr* disruptions on the identity, dendritic branching and dendritic spine morphologies of P17 cortical neurons.

(A) Forced expression of DENR WT does not significantly impair the long-term positioning of E14.5-born cells within the postnatal P17 cortex (2-way ANOVA F(4,50)=1.1, P=0.3583). (B) Treatment with *Denr* shRNA or forced expression of DENR WT, C37Y or P121L did not significantly affect the proportion of GFP+/CUX1+ cells in the cortex (1-way ANOVA F(4,10)=1.189, P=0.3732). (C to D) ANCOVA analysis of Sholl profiles to characterize potential differences in dendritic complexity of P17 cortical neurons. Graphs plot mean ± SEM. (C and D)

ANCOVA analysis of branching profiles between control (GFP-only; plotted with red triangles) treatment and *Denr* shRNA treatment (plotted with green circles) shows regional differences in dendritic branching. While there is no significant difference in the branching complexity of neurons between Sholl intersections analysed 5-25μm from the soma ($P = 0.456$), there is a significant difference in Sholl profiles analysed 30-50μm from the soma ($P < 0.05$) which indicates that *Denr* shRNA-treated neurons are more simplified within this domain. (E) Forced expression of DENR WT also leads to a significant difference in the Sholl profile 30-50μm from the soma when compared with control-treated neurons ($P < 0.05$).

Figure S4 related to Figure 4. Disruptions to DENR do not significantly alter the morphology of dendritic spines.

Dendritic spine types along the apical and basal dendrites, classified as filopodia, long thin, stubby, mushroom. There was no significant interaction between Denr perturbations and dendritic spine morphologies on apical dendrites (2-way ANOVA $F(6,176)=1.403$, $P=0.2158$) as well as basal dendrites (2-way ANOVA $F(6,196)=0.9675$, $P=0.4484$). Graphs plot mean ± SEM.

Figure S5 related to Figure 5. Studies of FLAG-DENR(WT), FLAG-DENR(C37Y) and FLAG-DENR(P121L) protein *in vitro* and *in vivo*.

(A) Multiple species alignments for DENR polypeptide sequences reveal that C37 is conserved between humans and drosophila, while P121 is not (key residues are identified in red text). (B) Quantification of immunoprecipitated myc-MCTS1 as immunoblotted signals relative to myc-MCTS1 input for biological triplicate experiments revealed no significant differences between DENR(WT), DENR(P121L)

and DENR(C37Y) (1-way ANOVA $F(2,6)=0.8183$, $P=0.485$, n=3 biological replicates). (C) Quantification of MYC-MCTS1 protein signals relative to β-actin suggests a trend in which transfection of DENR(C37Y) results in a reduction MYC-MCTS1 derived from co-transfected construct (1-way ANOVA $F(2,3)=7.731$, $P=0.065$, n=3 biological replicates). (D) Luciferase reporter assay using a heterologous human reporter. Multiple biological replicates indicate that hDENR(C37Y) consistently fails to rescue luciferase reporter activity (under the control of a heterologous human stuORF) as a result of siRNA-mediated loss of endogenous hDENR, whereas hDENR(P121L) gives highly variable results, suggesting a mild functional impairment. HeLa cells were treated with non-targeting control siRNA or siRNA targeting DENR, and then re-constituted with constructs expressing DENR(WT), DENR(C37Y) or DENR(P121L) which were refractory to siRNA-mediated silencing. (E) Immunostaining for FLAG-tagged DENR(WT), DENR(C37Y) and DENR(P121L) in E14.5-born cortical cells within the CP of E17.5 brains. The cellular distribution of FLAG immunoreactivity was not qualitatively different between DENR WT and its variants. Graphs plot mean ± SEM. Scale bar, (E) = 30µm.

Figure S6 related to Figure 6. Distinct effects of DENR C37Y and DENR P121L overexpression on the long-term positioning of cortical neurons, as well as their dendritic complexity.

(A) The migration profile of DENR C37Y overexpressing cells is different to DENR WT (2-way ANOVA $F(8,80)=3.1$, $P=0.0042$), since the migration profile of DENR C37Y treated cells is significantly different to Control, while DENR WT treated cells are not. (B) The migration profile of DENR P121L overexpressing cells is also

significantly different, albeit to a lesser degree. (C and D) Detection of heterotopic clusters of GFP-labelled cells within the electroporated brains of postnatal P17 animals following *Denr* disruption. The incidence of heterotopic clusters of GFP-labelled cells within each mouse brain was variable between treatment, and we found clusters either comprising GFP^{+}/$CUX1^{-}$ cells, or a mixture of GFP^{+}/$CUX1^{-}$ cells together with GFP^{+}/$CUX1^{+}$ cells (C). Representative images of heterotopic clusters of cells identified in two independent brain samples electroporated with DENR P121L (D). In one brain, a cluster of cells with a mixture of $CUX1^{+}$ and $CUX1^{-}$ cells was evident, while another brain sample comprised a cluster of GFP+ cells which did not co-label with CUX1. (E) Sholl analysis demonstrates that while forced expression of DENR WT enhanced the dendritic complexity of layer II/III neurons within the P17 cortex compared to control (GFP only), DENR C37Y and DENR P121L were impaired in their capacity to promote dendritic complexity since their Sholl profiles lie under the profile plot for DENR WT. (F to G) Differences in the local dendritic complexity of DENR C37Y and DENR P121L treated neurons compared to control (GFP only) neurons. The dendritic complexity of DENR C37Y-treated neurons is significantly different within Sholl intersections 40-65μm from the soma, while the dendritic complexity of DENR P121L-treated neurons is significantly different within Sholl intersections 30-50μm from the soma. (H to I) Differences in the local dendritic complexity of DENR C37Y but not DENR P121L treated neurons when compared to *DENR WT* neurons. The dendritic complexity of DENR C37Y-treated neurons is significantly different within Sholl intersections 25-45μm from the soma ($P < 0.05$), while the complexity of DENR P121L-treated neurons is not significantly different to DENR WT neurons at this location ($P = 0.153$). (J and K) Analysis of dendritic spine shapes (filopodia, long thin, stubby, mushroom) between control, DENR C37Y and

DENR P121L reveals no significant interaction between treatment for spine shapes on apical (2-Way ANOVA F(6,192=1.760, P=0.1092) and basal (2-Way ANOVA F(6,196=1.260, P=0.2775) dendrites. (L and M) The effects of mushroom-head spine volume and diameter following treatment with DENR C37Y or DENR P121L. Treatment with DENR P121L results in a significant decrease in the spine volume along apical dendrites (Control 0.3070±0.008899, DENR P121L 0.2565±0.01489, 1-way ANOVA F(2,514)=3.993, P=0.0190, Bonferroni multiple comparison *p<0.05) as well as along basal dendrites (Control 0.3185±0.01339, DENR P121L 0.2675±0.01138, 1-way ANOVA F(2, 451)=5.980, P=0.0027, Bonferroni multiple comparison *p<0.05). There was no significant effect of DENR C37Y or DENR P121L on the diameters of mushroom-shaped dendritic spines from apical (2-Way ANOVA F(2,514)=0.6026, P=5.478) and basal (2-Way ANOVA F(2,451)=3.062, P=0.0478) dendrites. Graphs plot mean ± SEM. Scale bar = (D), 200μm; boxed area, 44 μm.

Supplemental Table S1

**Table S1. Summary of Clinical Data in DENR mutation cases**		
	**Case 1 (Neale et al., 2012)**	**Case 2**
**Genetic mutation**	c.110G>A p.C37Y	c.362C>T p.P121L
**Family history**	No family history of ASD	Unrelated parents, first child, normal pregnancy and delivery
**Developmental delay**	Verbal IQ 60	Language delay
**Cognition**	Performance IQ 63<br>Full scale IQ 67	Poor comprehension, diagnosed Asperger's and depression
**Behaviour**	Autistic spectrum disorder	Asperger syndrome, depression
**MRI Brain**		Bilateral periventricular nodular heterotopia
**Other features**		Facial asymmetry, scoliosis, delayed walking (abnormally positioned feet)

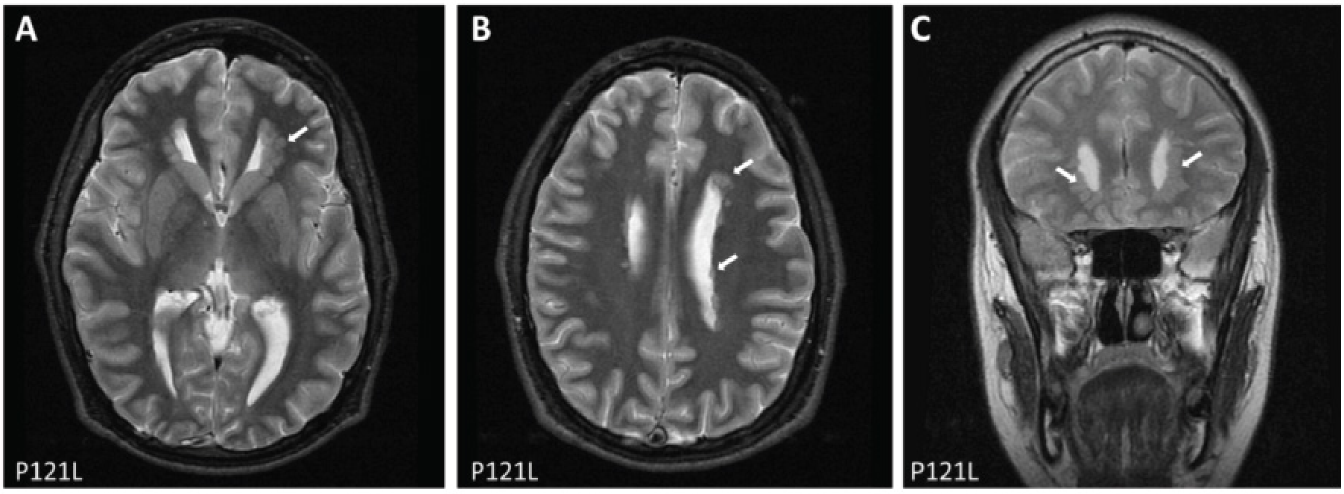

MRI images of Case 2, white arrows indicating paraventricular nodal heterotopia

Patient (Case 2)

Father

Mother

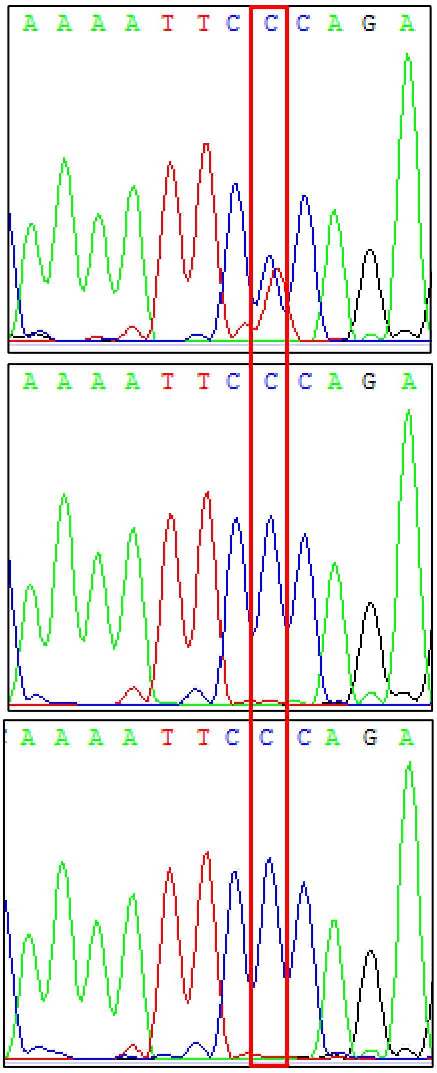

Translation Patient (Case 2)

AAA	ATT	CTC	AGA
Lys	Ile	Leu	Arg

Translation Parents

AAA	ATT	CCC	AGA
Lys	Ile	Pro	Arg

Supplemental Table S2

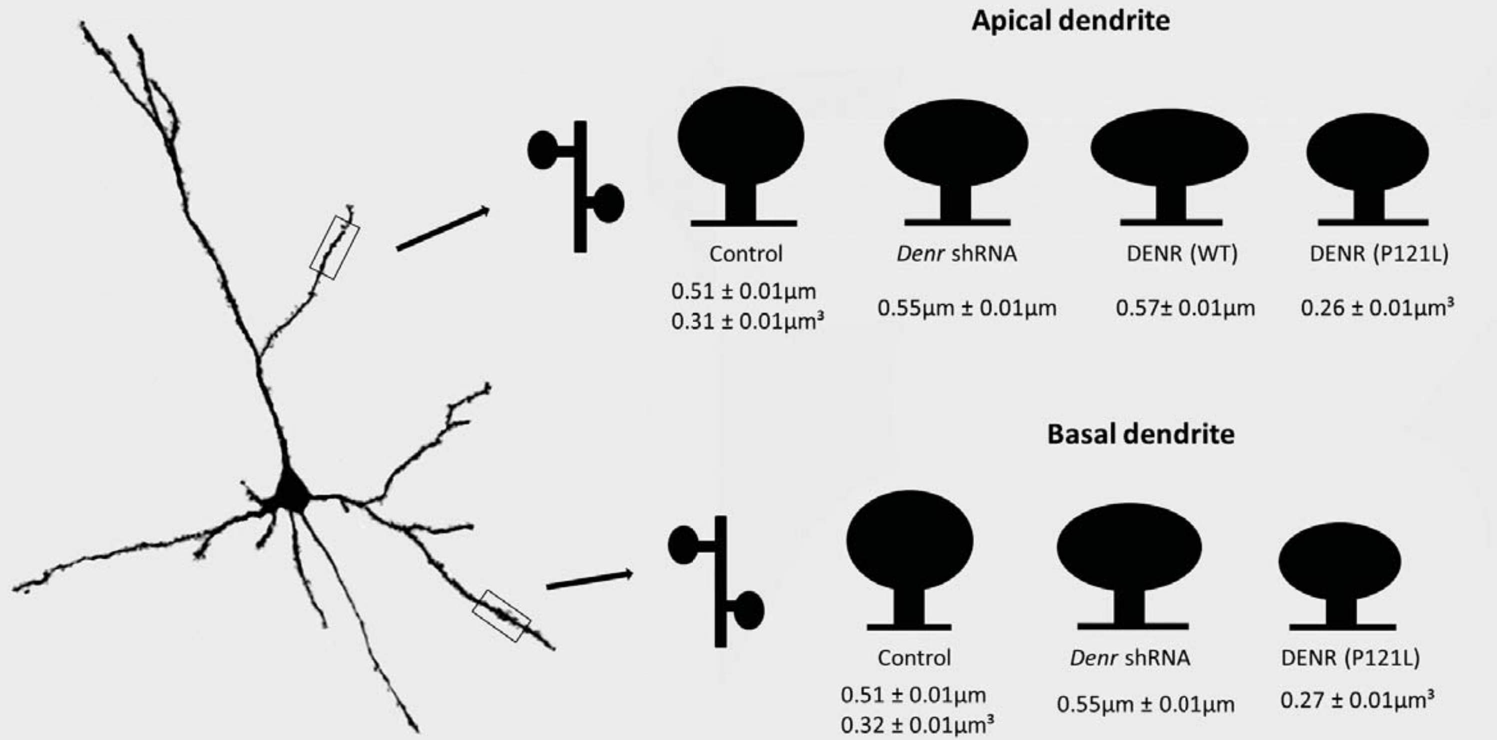

	Denr shRNA		DENR (WT)		DENR (C37Y)		DENR (P121L)	
	Apical	Basal	Apical	Basal	Apical	Basal	Apical	Basal
Spine Density	decreased	decreased	decreased	ns	decreased	decreased	decreased	decreased
Mushroom Spine Head Diameter	increased	increased	increased	ns	ns	ns	ns	ns
Mushroom Spine Head Volume	ns	ns	ns	ns	ns	ns	decreased	decreased

LEGENDS TO SUPPLEMENTARY TABLES

Supplemental Table S1. Summary of clinical and gene sequencing data in human cases of neurological disorders associated with mutations to *DENR*. Structural MRI imaging reveals paraventricular nodal heterotopia, a neuronal migration disorder. Sequence traces for a patient and their parents demonstrating a *de novo* heterozygous c.362C>T mutation resulting in a p.P121L substitution. See Experimental Procedures for further details related to data collection.

Supplemental Table S2. Summary of changes detected in mushroom-shaped dendritic spines of layer II/III projection neurons within the P17 mouse cortex reported in this study (related to Figure 4 and Figure 6, as well as Supplemental Figures S4 and S6). Treatment with *Denr* shRNA or overexpression of DENR WT led to a significant increase in the diameter (denoted "*d*") of spines, while overexpression of DENR P121L led to a significant increase in the volume (denoted "*V*") of spine heads. In basal dendrites, treatment with *Denr* shRNA similarly led to a significant increase in spine diameter, while overexpression of DENR P121L led to a reduction in the volume of spine heads.

SUPPLEMENTAL PROCEDURES

Additional cloning of a mouse cDNA expression construct (muDenr) refractory to shRNA-mediated silencing*

A mouse Denr cDNA flanked by MfeI restriction sites and comprising silent mutations within the targeting sequence recognized by *Denr* shRNA1 was synthesized by a commercial provider (IDT Technologies, USA) such that the

original target sequence 5’- CCAAGTTAGATGCGGATTA-3’ was mutated to 5’-CAAAGTTGGACGCCGATTA 3’ (underlined text represent modified nucleotide positions on Denr cDNA). The cDNA was cloned directionally into the EcoRI site of pCIG-F vector by conventional ligase-dependent cloning. The construct was verified by sequencing before an endolow midiprep was prepared for cell transfection experiments and *in utero* electroporation. *In utero* electroporation was peformed as described in Experimental Procedures. Immunostaining was performed using the following primary antibodies: rabbit anti-phosphorylated Histone-H3(ser10) (06-570, Merck Millipore, 1:1000), rabbit anti-Pax6 (PRB-278P, Covance, 1:500) and rabbit anti-Tbr2 (ab23345, Abcam, 1:500). Species-appropriate Alexa Fluor (Molecular Probes) secondary antibodies were used for immunofluorescence detection.

SUPPLEMENTAL REFERENCE

Consortium, F., the, R.P., Clst, Forrest, A.R., Kawaji, H., Rehli, M., Baillie, J.K., de Hoon, M.J., Haberle, V., Lassmann, T., et al. (2014). A promoter-level mammalian expression atlas. Nature 507, 462-470.
